# Integrated Gene Co-expression Analysis and Metabolites Profiling Highlight the Important Role of ZmHIR3 in Maize Resistance to *Gibberella* Stalk Rot

**DOI:** 10.3389/fpls.2021.664733

**Published:** 2021-05-11

**Authors:** Yali Sun, Xinsen Ruan, Qing Wang, Yu Zhou, Fang Wang, Liang Ma, Zhenhua Wang, Xiquan Gao

**Affiliations:** ^1^State Key Laboratory for Crop Genetics and Germplasm Enhancement, Nanjing Agricultural University, Nanjing, China; ^2^Jiangsu Collaborative Innovation Center for Modern Crop Production, Nanjing Agricultural University, Nanjing, China; ^3^College of Agriculture, Nanjing Agricultural University, Nanjing, China; ^4^College of Agriculture, Northeast Agricultural University, Harbin, China

**Keywords:** anthocyanin, co-expression network, cell death, *Gibberella* stalk rot, hypersensitive induced reaction 3, maize

## Abstract

*Gibberella* stalk rot (GSR) caused by *Fusarium graminearum* is one of the most devastating diseases causing significant yield loss of maize, and GSR resistance is a quantitative trait controlled by multiple genes. Although a few quantitative trait loci/resistance genes have been identified, the molecular mechanisms underlying GSR resistance remain largely unexplored. To identify potential resistance genes and to better understand the molecular mechanism of GSR resistance, a joint analysis using a comparative transcriptomic and metabolomic approaches was conducted using two inbred lines with contrasting GSR resistance, K09 (resistant) and A08 (susceptible), upon infection with *F. graminearum*. While a substantial number of differentially expressed genes associated with various defense-related signaling pathways were identified between two lines, multiple hub genes likely associated with GSR resistance were pinpointed using Weighted Gene Correlation Network Analysis and K-means clustering. Moreover, a core set of metabolites, including anthocyanins, associated with the hub genes was determined. Among the complex co-expression networks, *ZmHIR3* showed strong correlation with multiple key genes, and genetic and histological studies showed that *zmhir3* mutant is more susceptible to GSR, accompanied by enhanced cell death in the stem in response to infection with *F. graminearum*. Taken together, our study identified differentially expressed key genes and metabolites, as well as co-expression networks associated with distinct infection stages of *F. graminearum*. Moreover, *ZmHIR3* likely plays a positive role in disease resistance to GSR, probably through the transcriptional regulation of key genes, functional metabolites, and the control of cell death.

## Introduction

Plants are constantly encountering dangers from their living environment. Diseases caused by diverse phytopathogens are the major threats on crop production. As one of the most economically important crop species worldwide, maize (*Zea mays* L.) is naturally impacted by many types of diseases, among which the stalk rot caused by multiple pathogens, including *Fusarium verticillioides*, *Fusarium graminearum*, *Colletotrichum graminearum*, and *Pythium* spp., usually results in significant yield loss of maize. Moreover, the stalk rot also retards the extensive application of grain mechanical harvesting techniques (De León, 1984; Cheng et al., 2019). Particularly, *F. graminearum* has been well-known for its severe pathogenicity and aggressiveness on maize (Santiago et al., 2007; Jackson-Ziems et al., 2014), which causes not only *Gibberella* stalk rot (GSR) and *Gibberella* ear rot of maize, but also devastating diseases on other cereal crops, such as *Fusarium* head blight, crown rot, and seedling blight on wheat (*Triticum aestivum*) and barley (*Hordeum vulgare*) (Bai and Shaner, 2004; Goswami and Kistler, 2004; Jia et al., 2019). In addition, *F. graminearum* and *F. verticillioides* also contaminate maize and wheat grains by producing the carcinogenic mycotoxins, such as deoxynivalenol and fumonisins, respectively, which are extremely harmful to human beings and animals (Jia et al., 2009; Alberts et al., 2019).

To engage the successful penetration, colonization, and infection on host tissues, different phytopathogens have evolved distinct lifestyles to facilitate their pathogenicity. The majority of phytopathogens deploy either biotrophic or necrotrophic or both types, e.g., hemibiotrophic habits, to overcome resistance of host plants, by which biotrophic pathogens survive on the living tissues of host, whereas necrotrophic ones have to rely on the dead tissues by killing host cells (Zhu et al., 2020). However, hemibiotrophic pathogens, such as *F. graminearum*, have developed the strategy with separate phases, e.g., biotrophy at the initial stage, whereas necrotrophy at a later stage of infection, respectively (Glazebrook, 2005). A number of microscopic studies showed that, in line with its hemibiotrophic lifestyle, *F. graminearum* hyphae remained in the intercellular spaces upon penetration, causing cell death and necrosis in wheat riches, rather than destroying the cells before they proceeded to subsequent intracellular growth (Brown et al., 2010; Kazan et al., 2012). Another study using the fluorescence-labeled *F. graminearum* isolate to examine the infection process in maize stalk clearly showed that the hyphae grew intercellularly at the early infection stage, e.g., 24 h postinoculation (hpi), but intracellularly and intercellularly between 36 and 48 h, while the fungi fully occupied the invaded cells after 72 h, which was required for the virulence (Tian et al., 2016). A similar phenomenon was also observed in wheat coleoptiles using laser capture microdissection approach (Zhang X.W. et al., 2012). In supporting these findings, the expression levels encoding cell wall–degrading enzyme genes at earlier stage were up-regulated. On the contrary, reactive oxygen species and secondary metabolite biosynthesis-related genes were found to be induced at a later stage of infection, when obvious necrosis or cell death in host tissue was observed (Zhang X.W. et al., 2012). However, the mechanisms underlying host resistance to distinct infection stages of *F. graminearum* are not fully understood.

It has been well documented that immune responses triggered by the recognition of pathogen invasion by plants are often accompanied by hypersensitive response (HR)–like cell death, also known as programmed cell death (PCD), which is often considered as the hallmark of ETI response (Mukhtar et al., 2016; Huysmans et al., 2017; Jorgensen et al., 2017; Kabbage et al., 2017; Nagata and Tanaka, 2017; Gao et al., 2018; Pitsili et al., 2020). Multiple molecular components associated with PCD have been characterized. For instance, brassinosteroid-insensitive 1 (BRI1)–associated kinase 1 (BAK1) was broadly known to play roles in both development- and resistance-related PCD processes (Kemmerling et al., 2007; Gao et al., 2009; Gao et al., 2018), for which BAK1 might undergo the Ca^2+^-dependent proteolytic cleavage, likely by the protease calpain (Zhou J. et al., 2019). Moreover, multiple hypersensitive induced reaction (HIR) proteins, which are involved in proliferation and cell cycle control, ion channel regulation, and plant cell death (Nadimpalli et al., 2000), have also been extensively reported to play roles in mediating HR response and disease resistance, likely via associating with leucine-rich repeat (LRR) proteins. For example, OsHIR1 in rice was shown to be localized on plasma membrane and interacts with OsLRR1 to trigger HR response (Zhou et al., 2010). Similarly, pepper HIR1 protein also interacts with CaLRR1, to coordinate the HR process (Jung and Hwang, 2007). Despite this information, the roles of these components in GSR resistance are unknown.

GSR resistance is a quantitative trait controlled by multiple genes regulating dynamic networks, indicating the mechanism complexity mediated by resistance genes. Currently, several quantitative trait loci (QTLs) associated with GSR resistance have been successfully identified, and molecular mechanisms underlying resistance to GSR mediated by candidate genes for a few of these QTLs were investigated. For instance, *qRfg1* is a major GSR resistance locus on chromosome 10 (Yang et al., 2010), and *ZmCCT* was cloned as its causal gene (Wang et al., 2017a). Intriguingly, the epigenetic analysis demonstrated that the allelic variation caused by a polymorphic CACTA-like transposable element in the 2.4 kb upstream of *ZmCCT* resulted in its histone and subsequently the suppression of *ZmCCT* expression, which enables more precise and timely control of defense against GSR. Another QTL *qRfg2* encoding auxin regulatory protein ZmAuxRP1 was mapped to chromosome 1 (Zhang D. et al., 2012) and was shown to modulate GSR resistance and growth through balancing IAA content and benzoxazinoid biosynthesis (Ye et al., 2018). Moreover, another major QTL *qRfg3*, conferring the recessive resistance to GSR by ∼26.6%, was mapped into a 350-kb interval on chromosome 3 (Ma et al., 2017), yet no candidate genes were reported.

Notwithstanding the significant efforts mentioned above, the molecular mechanisms underlying the spatiotemporal differentiation of resistance response to *F. graminearum*, transiting from biotrophic to necrotrophic phase, are worthy to be systemically investigated. Furthermore, whether the host cell death is involved in the immunity to GSR remains largely unknown, particularly the key genes associated with the cell death control at distinct biotrophic and necrotrophic phases of *F. graminearum* invading maize stem tissues. To address these questions, in this study, the integrated analyses using RNA-seq and the Weighted Gene Co-expression Network Analysis (WGCNA), in combination with metabolomics, were deployed to investigate the genome-wide transcriptional and metabolomic changes between a resistant and a susceptible maize line upon infection with *F. graminearum*. Multiple key differentially expressed genes (DEGs) and co-expression networks regulated by hub genes are identified using Kyoto Encyclopedia of Genes and Genomes (KEGG), Gene Ontology (GO) clustering, and WGCNA, which are likely associated with GSR resistance in a spatial pattern. Among these networks, *ZmHIR3* was accentuated for its strong co-expression with multiple defense-related pathways within the network. Phenotypic analysis showed that while *zmhir3* mutant became more susceptible to GSR, stronger cell death was observed in the stem of mutant compared to wild-type (WT) upon infection with *F. graminearum*, suggesting that cell death coordinated by *ZmHIR3*-centered signaling complex plays an important role in modulating the resistance to GSR.

## Materials and Methods

### Plant Material and Growth Conditions

A mini natural population consisting of 66 maize inbred lines was collected from different resources. The mutant of *ZmHIR3* (ID: Zm00001d039173), *zmhir3*, was generated through ethyl methane sulfonate (EMS) mutagenesis in B73 background, and the mutation in M1 seeds was detected by whole-exome capture and next-generation sequencing (Lu et al., 2017). M3 seeds were propagated and used in this study. The single-nucleotide polymorphism (SNP) mutation of mutant was confirmed by resequencing using a pair of primers flanking about 200 bp upstream and downstream of the mutation site. The primers are listed in Supplementary Figure 1.

For seedling assays, the seeds were sown in the soil in long pots made with PVC cylinders (5 cm in diameter and 20 cm in depth), with 2 seeds per cylinder. The pots were maintained on plant growth shelves under controlled conditions, a temperature at 26°C ± 2°C during a 14/10-h light–dark cycle, with the light density of 600 Lux, and humidity of approximately 75%. The seedlings in similar size at approximately 12-d to 2-week-old stage were selected and transferred to a culture room for phenotype assay.

The seeds of *Nicotiana benthamiana* were grown at 23°C with a cycle of 16-h light and 8-h dark, until the use for transient transformation.

### Artificial Inoculation and Disease Phenotype Evaluation

*Fusarium graminearum* strain 0609, kindly provided by Prof. Mingliang Xu at China Agricultural University, was cultured and maintained on PDA media as regular. The fungal inoculum and artificial inoculation on seedlings were conducted according to our previous study (Sun et al., 2018). Briefly, the fungus was cultured freshly in liquid common bean soup for 2 to 3 days, and fungal culture was centrifuged, resuspended, and washed twice with sterile water, and then the spore suspension was examined under microscope and adjusted to a concentration of 1.0 × 10^6^/mL in 0.001% Tween-20. The seedlings were horizontally laid down in a plastic square tray (100 × 80 × 10 cm) in a controlled incubation room (light cycle and temperature: 14-h light/10-h dark at 24°C ± 2°C; light intensity at 200 Lux), and 20 μL of spore suspensions was inoculated to the wounded point carefully on the stem. The disease symptoms were scored at 3 days postinoculation (dpi), by a 5-level scale ranging from 1 (most resistant) to 5 (most susceptible). The disease symptom index (DSI) was calculated according to the formula described by Ma et al. (2017) with slight modification: DSI (%) = Σ(ranking scale × number of plants at that rank) × 100/(5 × total number of plants) (Tanaka et al., 2014). The statistical analyses were performed using SPSS (14.0) software for analysis of variance (ANOVA) and the Student *t*-test, with the level of significance set at *p* < 0.05.

### RNA-Seq Transcriptome Analysis and Quantitative Reverse Transcription–Polymerase Chain Reaction Validation

RNA was extracted from the seedling stems at different time points inoculated with F. *graminearum* using TRIzol reagent (Invitrogen) and then purified with the RNeasy Mini Kit (Qiagen). To better understand the global gene expression pattern associated with GSR resistance by comparison of two lines with contrasting phenotype, we refer the strategy of previous similar work on RNA sampling and sequencing approaches from maize samples upon *Fusarium* infection (Liu et al., 2016; Zhang et al., 2016). The stems inoculated with 0.001% Tween-20 upon wounding was used as control. RNA samples were subject to quality control prior to the library construction using Illumina TruSeq RNA library prep kit v2. Subsequently, the RNA sequencing was performed using Illumina HiSeq 2000 (Illumina Inc.) at Berry Company, Beijing, China.

Paired-end RNA sequence reads of 150 bases were generated, and all clean reads from sequencing were aligned to maize inbred B73 reference genome (RefGen_V4) and the reference gene model dataset of *F. graminearum* (FGS 5b) using hisat2. The gene expression value was normalized as gene counts with Stringtie 1.3.5. The differently expressed genes were produced with the threshold of a false discovery rate (FDR) < 0.05 by the DESeq software Packages^1^. Genes were annotated based on maize genome from NCBI. The identified DEGs were subjected to GO enrichment and KEGG pathway analyses with R package clusterProfiler 3.10.1 as described previously, respectively (Ashburner et al., 2000; Yu et al., 2012; Kanehisa et al., 2015). Three independent biological replicates were conducted with each sample containing at least six seedlings.

To validate the RNA-seq data, quantitative reverse transcription–polymerase chain reaction (qRT-PCR) analysis was performed on randomly selected representative genes using the AceQ qPCR RT SYBR Green Master Mix (Q212-01, Vazyme) in a Bio-Rad real-time instrument (Roche PCR-480). The PCR program consisted of an initial denaturation step at 95°C for 5 min, followed by 40 cycles of 95°C for 10 s, 55°C–60°C (depending on primers’ annealing temperature) for 30 s, and 95°C 15 s, 60°C 1 min, and 95°C 15 s. The relative expression levels were calculated using 2^–Δ^ ^Δ^ ^*Ct*^, and the transcript level of *ZmActin* (Zm00001d01227) was used for internal normalization. All qRT–PCR reactions were performed with three biological replicates for each sample. qRT-PCR primers are listed in Supplementary Table 1.

### Weighted Gene Co-expression Network Analysis and Network Construction

RAW RNA-seq count data were processed and normalized using the function “variance StabilizingTransformation” of R package Deseq2 (Love et al., 2014), and 32336 genes were selected using function “goodSamplesGenes” of R package WGCNA 1.68 (Langfelder and Horvath, 2008), and then a soft threshold of 8 was selected using the function “pickSoftThreshold” as it shows the independence degree above 0.9 with minimum power value. With the chosen soft threshold value, modules were conducted and processed with dynamic branch cutting with a merging cutoff threshold of 0.25, and 27 modules in total were obtained.

To evaluate modules correlated with the *F. graminearum* infection, the ratio of RNA-seq reads mapping to the *F. graminearum* genome was calculated and taken as sample trait, and then the correlation between module eigengenes and trait was analyzed. The eigengenes of seven modules (turquoise, blue, pink, green, black, dark turquoise, dark gray) significantly correlated with the reads of *F. graminearum* were selected. Based on the cutoff criteria (| module membership| > 0.8 and | gene significance| > 0.2), genes with high connectivity in the significant module were identified. Correlation analyses were performed using the method of “Pearson” as described in WGCNA guides.

### K-Means Clustering Analysis

Genes in WGCNA modules were clustered with R “kmeans” method, based on the Log2 fold-change value at 6–12–24 hpi (early stages) and 48–72 hpi (later stages), respectively, and the number of clusters was set as 25 based on the value within groups’ sum of squares. To determine the clusters that showed different expression patterns between resistant and susceptible samples, variation of Log2 fold-change value was calculated in resistant samples, susceptible samples, and all samples, respectively, and clusters with high ratio of average variation between resistant and susceptible; all samples were filtered (ratio threshold set as 2), and genes in these cluster were selected and used for further analysis.

### Metabolomic Analysis

The sample preparation and metabolomic analysis were provided by MetWare Company (Wuhan, China), according to the manufacturer’s protocols, with some modifications. The freeze-dried stem tissues were crushed, and 100 mg powder was weighted and extracted overnight at 4°C with 0.6 mL 70% aqueous methanol. Following centrifugation at 10,000*g* for 10 min, the extracts were absorbed by CNWBOND Carbon-GCB SPE Cartridge (250 mg, 3 mL) and filtered using SCAA-104 Cartridge (0.22-μm pore size) (ANPEL, Shanghai, China^2^). The samples extracted were subsequently analyzed using an ultraperformance liquid chromatography–electrospray ionization (ESI)–tandem mass spectrometry (MS/MS) system (Shim-pack UFLC SHIMADZU CBM30A system^3^; MS, Applied Biosystems 4500 Q TRAP^4^). The effluent was alternatively subjected to an ESI-triple quadrupole-linear ion trap (QTRAP)–MS on an API 4500 Q TRAP UPLC–MS/MS system, equipped with an ESI turbo ion-spray interface, operating in positive and negative ion modes and controlled by Analyst 1.6.3 software (AB Sciex).

Unsupervised PCA (principal component analysis) was performed by statistics function prcomp within R^5^. The data were unit variance scaled before unsupervised PCA. The HCA (hierarchical cluster analysis) results of samples and metabolites were presented as heatmaps with dendrograms, whereas Pearson correlation coefficients (PCCs) between samples were calculated by the cor function in R and presented as only heatmaps. Both HCA and PCC were carried out by R package pheatmap. For HCA, normalized signal intensities of metabolites (unit variance scaling) are visualized as a color spectrum.

Significantly regulated metabolites between two genotypes during infection period were determined by two-way ANOVA, and the heatmaps and major change patterns were generated using MetaboAnalyst 5.0. Identified metabolites were then annotated using KEGG Compound database^6^ and then mapped to KEGG Pathway database^7^. Pathways mapped with significantly regulated metabolites were then fed into MSEA (metabolite sets enrichment analysis), and the significance of differentially accumulated metabolites (DAMs) was determined by hypergeometric test’s *p*-values.

### Correlation Analysis Between Metabolome and Transcriptome Data

Pearson correlation coefficients was calculated for metabolome and transcriptome data integration. In this study, Log2 conversion of data was performed uniformly before analysis. For the joint analysis between the metabolome and transcriptome, cor program from R was used in this experiment, and the screening criteria were PCC > 0.8. The relationships between metabolome and transcriptome data were visualized by using Cytoscape (The Cytoscape Consortium, San Diego, CA, United States, version 3.7.0).

### Plasmid Construction and Transient Transformation Using *Agrobacterium tumefaciens*

To identify the subcellular localization of ZmHIR3, the coding sequences (CDS) of ZmHIR3 were amplified and cloned into the binary vector pCambia1305.1-GFP by restriction enzymes *Spe*1 and *Bam*H1 using homologous recombination method. The primers used for cloning are listed in Supplementary Table 1.

The detailed procedures of *Agrobacterium tumefaciens*–mediated transient expression were performed according to previous study with some modifications (Wang et al., 2015). *A. tumefaciens* strain GV3101 transformed with plasmid ZmHIR3-1305.1-GFP was grown at 28°C in LB broth medium supplemented with 50 μg/mL kanamycin and rifampicin overnight, respectively, and then resuspended in the infiltration buffer [10 mM MgCl_2_, 10 mM MES (pH 5.6), and 150 μM acetosyringone] and kept in dark at room temperature for 3 h. *A. tumefaciens* culture with an OD600 = 1.0 was then infiltrated into the *N. benthamiana* leaves, which were detached at 72 h postinfiltration, and the protein subcellular localization was examined under a laser confocal microscope. *A. tumefaciens* transformed with empty binary vector was used as a control.

### Detection of Cell Death in Maize Stem Infected With *F. graminearum*

Cell death in maize stems upon infection with *F. graminearum* was observed using trypan blue staining method with some modification (Wu et al., 2020). The stems of 2-week-old seedlings of A08 and K09 after inoculation with *F. graminearum* were excised at 1 cm above and below the inoculation site and subsequently immersed into a 15-mL centrifuge tubes containing 10 mL of 0.3 mg/mL of trypan blue solution dissolved in lactophenol (lactic acid:glycerol:liquid phenol:distilled water 1:1:1:1) and then incubated in boiling water bath for 3 min. The stems were then decolored in 0.15% of trichloroacetic acid dissolved in ethyl alcohol:trichloromethane (75:25, vol/vol) at room temperature for 24 h. The outer layer of the stem was peeled, mounted in 50% glycerol, and visualized under a microscope. All experiments were performed with at least three biological replicates with consistent results obtained.

## Results

### Identification of a Pair of Maize Lines With Contrast GSR Phenotype

**FIGURE 1:**
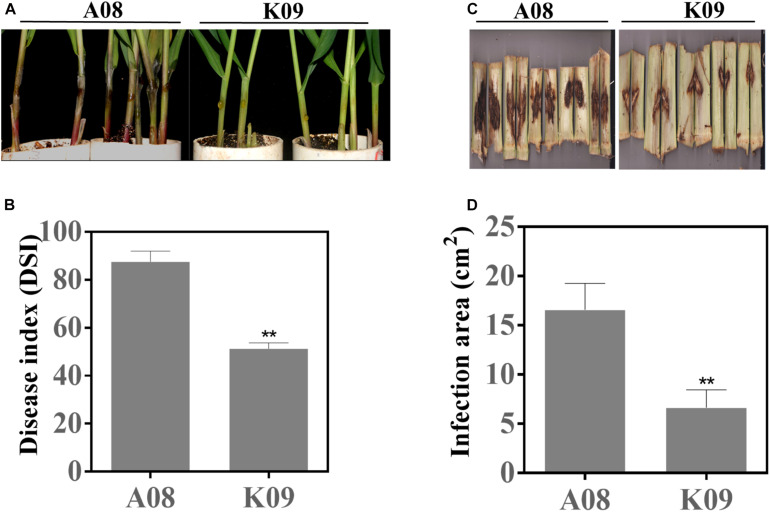
GSR phenotype of susceptible line A08 and resistant line K09 upon infection with *Fusarium graminearum*. **(A)** Seedling GSR phenotypes of A08 and K09. The phenotype was recorded at 3 dpi. **(B)** Quantification of seedling GSR disease index (DSI) of A08 and K09 shown in **(A)**. **(C)** Field GSR phenotype of A08 and K09 recorded at 15 dpi. **(D)** GSR disease areas on the stems of A08 and K09 in the field condition. Both seedling assay and field assay were conducted for at least three replicates with consistent results obtained, with at least 10 plants per replication. **Statistically significant (*p* < 0.01) between mock and inoculation treatments analyzed by *t*-test.

To identify GSR-resistant lines, we assessed the disease phenotypes of a maize population consisting of 66 inbred lines using the seedling assay as previously described (Sun et al., 2018). As shown in Supplementary Figure 1, the majority of lines showed the moderate disease severity to GSR, whereas several lines displayed obvious resistance (such as CML496, Zhong106, CIMBL133, etc.) or susceptibility (such as GEMS42, GEMS27, Z2018F, etc.) to GSR. To confirm the phenotypes in the field, we utilized the artificial infection method, by injecting *F. graminearum* suspension spores into the stem of these lines at flowering stage (Supplementary Figure 2A). The population in the field also showed obvious variation of disease severity levels, with the disease area ranging from approximately 3 to 21 cm^2^ (Supplementary Figure 2B). To compare GSR phenotypes of adult plants in the field and seedlings from laboratory, we conducted correlation coefficient analysis for 41 inbred lines that showed phenotypes between two set of samples. All plants were divided into three groups, all lines (All), lines with medium resistance and medium susceptibility (MR + MS), and lines with high resistance and high susceptibility (HR + HS). It showed that there were very low correlation coefficient (0.0289) for group All, with *p* = 0.8576, and negative correlation coefficient (-0.6022) for group MR + MS with *p* = 0.0018, respectively (Supplementary Table 2). On the contrary, group MR + MS displayed high correlation coefficient (0.7778) with *p* = 0.0002, indicating the consistence of GSR phenotype of lines with extreme phenotype between field assay and seedling assay (Supplementary Table 2). Among those materials, two lines with contrast phenotype, e.g., the resistant line W438 (ID: K09) and susceptible line 335M (ID: A08), exhibited very stable and consistent phenotypes across all replicates in both assays. As shown in Figure 1, most individual plants of A08 seedlings had obviously massive and white hyphae over the inoculation site, and the tissue became rotten with dark brown color, whereas K09 seedlings showed invisible hyphae around the injection site, with strong and upright stems (Figure 1A). The quantification of disease index showed that K09 had a significantly lower DSI at approximately 50, compared to that of A08 around 88 (Figure 1B). The field assay clearly showed that A08 had significantly larger lesion on the stem as compared to K09 (Figure 1C), the former with the average lesion size at 16.3 cm^2^, whereas the later at 6.25 cm^2^ (Figure 1D). Thus, these two lines displayed obviously contrast GSR resistance levels and were selected for further study.

### RNA-Seq Analysis of A08 Versus K09 Upon Infection With *F. graminearum*

To identify genome-widely the key genes associated with the resistance to GSR, we selected a pair of lines with contrasting GSR phenotype and conducted the RNA-seq analysis using A08 and K09 at 0 (control), 6, 12, 24, and 48 hpi with *F. graminearum*. As shown in Supplementary Table 3, 26.1 million reads on average of up to 150 bases in length were obtained from each sample, with 26.7 million for A08 and 25.6 million for K09, after filtering and quality control of the raw reads. The proportions of reads aligned to B73 reference genome (version 4.0) were approximately similar for both A08 (88.94%) and K09 (89.56%), suggesting a high correlation and reliability of sequencing data.

The sample-to-sample correlation analysis was then used for data analysis. The overall correlation between different samples at different time points was determined without a cluster dendrogram generated for A08 and K09 (Supplementary Figures 3A,B), which showed clearly that all the three biological replicates at a given time point were grouped together with high correlation and reliability for each sample, respectively. Interestingly, whereas the expression data at 6, 12, and 24 h were closely correlated together with CK, those at 48 and 72 h were clustered together exclusively in both lines (Supplementary Figure 3).

### Identification of DEGs Between Two Lines

**FIGURE 2:**
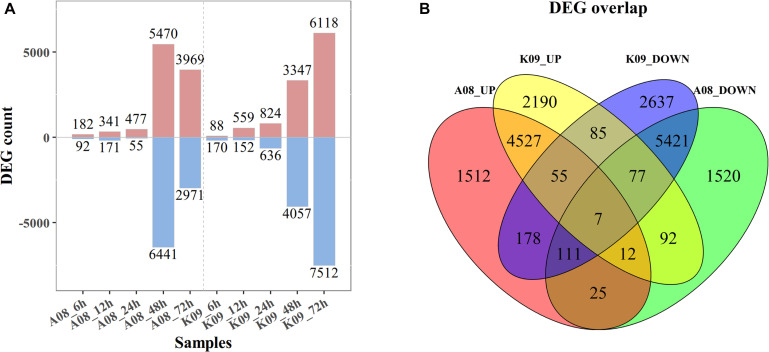
RNA-seq analysis of K09 and A08 upon infection with *F. graminearum*. **(A)** The total number of up- and down-regulated differentially expressed genes (DEGs) at 6 h postinoculation (hpi), 12, 24, 48, and 72 hpi in A08 and K09, respectively, after inoculation with *F. graminearum*. **(B)** The Venn diagram displaying differentially expressed transcripts unique and common between K09 and A08. The number of up-regulated and down-regulated genes is indicated by red and blue fonts, respectively.

To identify the DEGs between two contrast lines, an FDR of 0.05 was used as the cutoff value for statistical significance, and a fold change (FC) ≥ 2 of relative expression levels was used as the cutoff for FCs. As shown in Figure 2A, 182, 341, 477, 5,470, and 3,696 up-regulated DEGs were identified in A08 at 6, 12, 24, 48, and 72 hpi, whereas 88, 558, 824, 3,347, and 6,118 DEGs were found in K09, respectively. There were relatively fewer down-regulated DEGs than up-regulated at 6, 12, and 24 hpi in both lines, with 92, 171, and 55 in A08, 170, 152, and 636 in K09, respectively. However, more down-regulated DEGs than up-regulated were identified in both lines at later times (48 and 72 hpi), except that at 72 hpi in A08 (2971 DEGs). Clearly, there were significantly higher number of DEGs at later stages (48 and 72 hpi) than that at earlier stages (6, 12, and 24 hpi) in both lines, which is in line with the correlation analysis as shown in Supplementary Figure 3, indicating the distinctness of host defense response between the early and later stages in response to fungal infection.

To further determine the DEGs associated with the resistance, the unique and common DEGs between K09 and A08 were analyzed. As shown in Figure 2B, there were 1,512 unique DEGs significantly up-regulated in A08, whereas 2,190 in K09; 1,520 down-regulated DEGs were identified only in A08, whereas 2,637 only in K09. Moreover, there were 178 DEGs up-regulated in A08 but down-regulated in K09, and 92 DEGs up-regulated in K09 but down-regulated in A08. In addition, 4,601 DEGs were found to be up-regulated, and 5,616 DEGs down-regulated commonly in both lines (Figure 2B).

### GO and KEGG Analysis of DEGs

**FIGURE 3:**
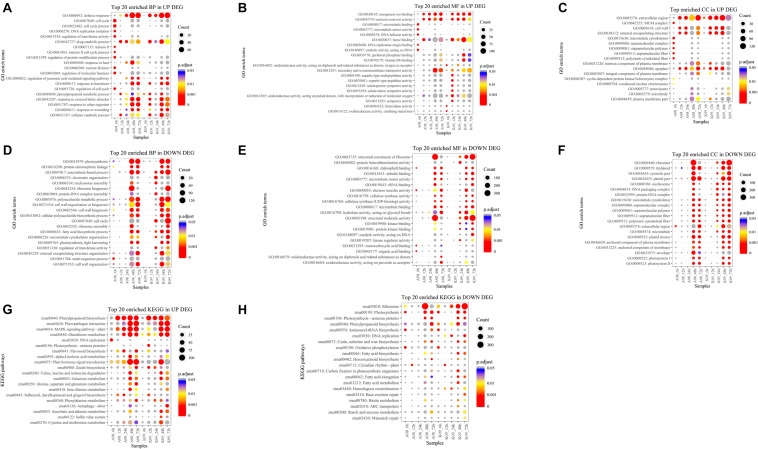
GO term enrichment **(A–F)** and KEGG analysis **(G,H)** of DEGs identified by RNA-seq. **(A)** Top 20 GO terms in up-regulated DEGs significantly enriched in biological process (BP). **(B)** Top 20 GO terms in up-regulated DEGs significantly enriched in molecular function (MF). **(C)** Top GO terms in up-regulated DEGs significantly enriched in cellular compartment (CC). **(D)** Top 20 GO terms in down-regulated DEGs significantly enriched in BP. **(E)** Top 20 GO terms in down-regulated DEGs significantly enriched in MF. **(F)** Top GO terms in down-regulated DEGs significantly enriched in CC. **(G)** Top 20 significantly enriched KEGG pathways in up-regulated DEGs. **(H)** Top 20 significantly enriched KEGG pathways in down-regulated DEGs. The size of black dot indicates the number of gene counts. The DEGs were selected with a criterion at adjusted *p*-value (*p.adj*) < 0.05 shown in colored side bar.

To determine the function of identified DEGs, GO analysis was carried out, and all DEGs were categorized into two functions, biological process (BP) and molecular function (MF). The abundance of each assembled transcript sequence in different samples was measured through normalized count (base mean) (Klein et al., 2017), and DEGs were defined as genes that were significantly increased or decreased in their expression levels with *p* < 0.05 and log2 FC ≥ 1.5. Overall, 18,449 DEGs were enriched in 215 GO terms, among which the top 20 GO terms up-regulated and down-regulated in BP, MF, and cellular component (CC) were selected based on the significance, respectively (Figures 3A–F). The top 20 significant GO terms in DEGs up-regulated in BP, which were significantly enriched at an early time (6 hpi) in susceptible line A08 compared to that in K09, mainly including “cell cycle” (GO:0007049), “cell cycle process” (GO:0022402), “DNA replication initiation” (GO: 0006270), “regulation of transferase activity” (GO:0051338), “meiosis II” (GO: 0007135), “meiosis II cell cycle process” (GO:0061983), “regulation of protein modification process” (GO:0031399), “nuclear division” (GO:0000280), “regulation of molecular function” (GO:0065009), and “regulation of cell cycle” (GO: 0051726). On the contrary, “defense response” (GO: 0006952), “response to bacterium” (GO: 0009617), “response to other organism” (GO:0051707), and “response to external biotic stimulus” (GO:0043207), are mainly significantly enriched starting at 12 hpi in resistant line K09, whereas at late time points, e.g., 24 and 48 hpi in susceptible line A08 (Figure 3A). In MF category, five GO terms, “microtubule binding” (GO:0008017), “microtubule motor activity” (GO:0003777), “DNA helicase activity” (GO:0003678), “DNA replication origin binding” (GO:0003688), and “catalytic activity, acting on DNA” (GO:0140097) within the top 20 significant GO terms, were significantly enriched in A08 but not K09 (Figure 3B). Inversely, eight GO terms related to immunity, including “oxidoreductase activity, acting on diphenols and related substances as donors, oxygen as receptor” (GO:0016682), “aspartic-type endopeptidase activity” (GO:0004190), “aspartic-type peptidase activity” (GO:0070001), “oxidoreductase activity, acting on paired donors, with incorporation or reduction of molecular oxygen” (GO:0016705), “solute: proton symporter activity” (GO:0015295), “solute: cation symporter activity” (GO:0015294), “symporter activity” (GO:0015293), and “ferroxidase activity” (GO:0004322), were significantly enriched in K09 but not A08 (Figure 3B). Regarding enriched CC in up-regulated DEGs, GO terms “MCM complex (GO:0042555),” “microtubule cytoskeleton” (GO:0015630), “supramolecular complex” (GO:0099080), “supramolecular polymer” (GO:0099081), “supramolecular fiber” (GO:0099512), and “polymeric cytoskeletal fiber” (GO:0099513) were specifically induced in A08 compared to K09 at 6 hpi, whereas “intrinsic component of plasma membrane (GO:0031226)” and “plasma membrane part” (GO:0044459) were enriched at an early time point (6 hpi) in K09 compared that at later time points (48 and 72 hpi) in A08 (Figure 3C).

On the contrary, several growth-related GO terms significantly enriched in BP were found to be down-regulated in K09 at 24 hpi, such as “microtubule-based process” (GO:0007017), “chromatin organization” (GO:0006325), “nucleosome assembly” (GO:0006334), “protein-DNA complex assembly” (GO:0065004), “cell cycle” (GO:0007049), “fatty acid biosynthetic process” (GO:0006633), “microtubule cytoskeleton organization” (GO:0000226), and “regulation of transferase activity” (GO:0051338) (Figure 3D). Similarly, although fewer DEGs significantly enriched in MF were found to be down-regulated, compared to that in BP, several GO terms, including “protein heterodimerization activity” (GO:0046982), “hydrolase activity acting on glycosyl bonds” (GO:0016798), “structural molecular activity” (GO:0005198), “kinase binding” (GO:0019900), “protein kinase binding” (GO:0019901), and “kinase regulator activity” (GO:0019207), were down-regulated in K09 at 24 hpi (Figure 3E). Interestingly, two GO terms “structural constituent of ribosome” (GO:0003735) and “structural molecule activity” (GO:0005198) were significantly enriched at 48 hpi in A08, whereas at 72 hpi in K09. Within top 20 enriched down-regulated GO terms in CC category, multiple terms also showed similar patterns, significantly enriched earlier (24 hpi) in K09 than A08, such as “nucleosome” (GO:0000786), “DNA packaging complex” (GO:0044815), “protein-DNA complex” (GO:0032993), etc. (Figure 3F). Taken together, growth-related genes were induced faster and greater in susceptible line A08, whereas defense-related genes in resistant line K09.

To better understand the biological function of DEGs between two lines upon infection with *F. graminearum*, we conducted an enrichment analysis with the KEGG pathway, and top 20 significantly enriched KEGG either up-regulated or down-regulated were selected based on the significance with adjusted *p* > 0.05. As shown in Figure 3G, several pathways including “Phenylpropanoid biosynthesis” (zma00940), “Plant-pathogen interaction” (zma04626), “MAPK signaling pathway-plant” (zma04016), “Glutathione metabolism” (zma00480), and “Plant hormone signaling transduction” (zma04075) with more DEGs in both lines were identified, especially at a later stage of infection (48 and 72 hpi). Among these pathways, “MAPK signaling pathway-plant” (zma04016) and “Glutathione metabolism” (zma00480) seemed to be enriched in A08 much earlier than in K09 at 12 hpi. Moreover, “DNA replication” (zma03030), “Flavonoid biosynthesis” (zma00941), and “beta-Alanine metabolism” were enriched more significantly in A08, whereas “Zeatin biosynthesis” (zma00908), “Autophagy” (zma04136), “Ascorbate and aldarate metabolism” (zma00053), and “Cysteine and methionine metabolism” pathways were found to be enriched in K09 (Figure 3G). Furthermore, fewer down-regulated DEGs were found to be significantly enriched both lines, whereas more significantly enriched pathways seemed to be associated with the later stages, e.g., 48 and 72 hpi, in K09, such as “ribosome” (zma03010), “DNA replication” (zma03030), “Fatty acid biosynthesis” (zma00061), “Fatty acid elongation” (zma00062), and “Fatty acid metabolism” (zma01212) (Figure 3H). To identify potential genes associated with resistance to GSR, we identified the DEGs in pathways specifically associated with disease resistance, including defense response, response to fungus, hydrogen peroxide catabolic process, response to jasmonic acid, and cell death (Supplementary Figure 4), respectively. It clearly showed that most of the DEGs in these pathways were induced significantly at later stages (e.g., 48 and 72 hpi) rather than earlier stages (6, 12, and 24 hpi). Specifically, “*probable L-type lectin-domain containing receptor kinase S.*7 (103629992)” and “*barley mlo defense gene homolog 3* (*542508*)” are highly induced at 6 hpi in A08, whereas “*lectin receptor kinase 7* (*100281599*),” “*thaumatin like protein 1* (*100283065*),” “*ZIM transcription factor* (*100384222*),” “*protein LAZ1* (*100502274*),” “*MACPF domain containing protein NSL1* (*103636097*),” “*protein argonaute 7* (*103636643*),” and “*L-type lectin-domain containing receptor* (*103639802*)” were up-regulated at later stages, mainly 48 and 72 hpi, which were significantly higher than those in K09, suggesting their possible involvement in the susceptibility to GSR (Supplementary Figure 4A). On the contrary, multiple genes, including “*Osmotin-like protein OSM34*,” “*terpene synthase 7* (*100281754*),” “*protein TIFY 11c* (*103626248*),” “*L-type lectin-domain containing receptor* (*103651308*),” and “*hypersensitive induced reaction 3* (*541872*)” (*HIR3*), were strongly induced in the resistant line K09, mainly at later stages (48–72 hpi) (Supplementary Figure 4A), suggesting the positive role of these genes in resistance to GSR.

Moreover, GO term “response to fungus” (0009620) contains 61 DEGs. In this category, whereas majority of DEGs were altered at a later stage (48 and 72 hpi) in both lines, several genes were significantly up-regulated in K09 but not in A08 at early time points (12, 24 hpi), such as “*terpene synthase 1* (*541974*),” “*stress-induced protein 1* (*542299*),” “*hevein-like preproprotein* (*100191593*),” “*pathogenesis protein 10* (*100192117*),” “*pathogenesis-related protein 1* (*103634525*),” and “*barwin* (*103652814*)” (Supplementary Figure 4B). Furthermore, GO term “hydrogen peroxide catabolic process” contains a set of peroxidase genes, the majority of which were highly up-regulated in K09 at a later time point (48 and 72 hpi), compared to that in A08, except “*peroxidase 2* (10027011),” “*peroxidase 2-like* (103635393),” “*peroxidase 3* (542505),” and “*peroxidase* (542571)” (Supplementary Figure 4C). Furthermore, several genes in cluster GO term “response to jasmonic acid,” such as “*IAA-amino acid hydrolase ILR1-like 6* (10019730),” “*jasmonic acid amido synthetase JAR1* (100381512),” and “*ZIM transcription factor* (100384222)” are strongly induced to higher level, except one gene, “*putative homeodomain-like transcription factor superfamily protein* (103638651)” at a later stage, which was activated in K09 (Supplementary Figure 4D). Similarly, GO term cluster “cell death,” “*protein BONZAI 3*” (100381409), “*protein LAZ1*” (100502274), and “*MACPF domain containing protein NSL1*” (103630717) were all up-regulated stronger and faster at 48 hpi in A08 than K09 (Supplementary Figure 4E).

### Validation of RNA-Seq by Real-Time qRT-PCR

To validate the reliability of RNA-seq data, six genes were randomly selected from different treatments for qRT-PCR analysis (Supplementary Figure 5). The expression pattern of genes quantified by qRT-PCR was similar to that by RNA-Seq. Thus, the reliability of RNA-seq used to identify DEGs associated with GRS phenotypes in this study was verified.

### Gene Co-expression Network Construction Using WGCNA

**FIGURE 4:**
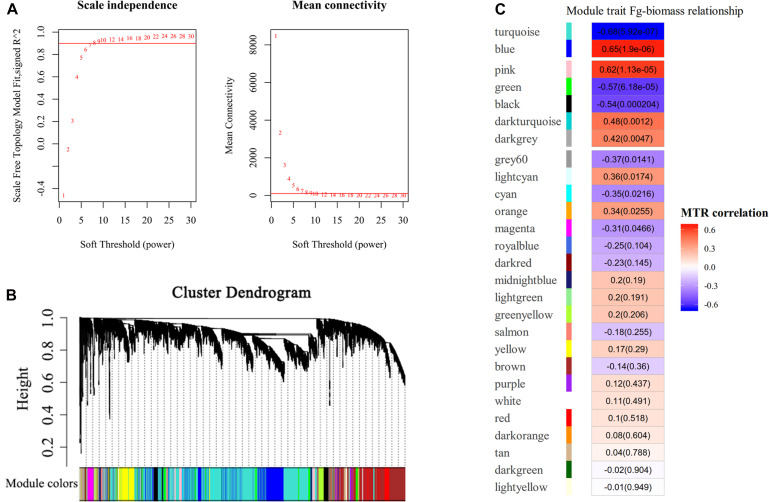
WGCNA analysis of RNA-seq data. **(A)** Analysis of network topology for different soft-thresholding powers. The left panel indicates the influence of soft-thresholding power (*x*-axis) on scale-free fit index (*y*-axis), and the right panel shows the influence of soft-thresholding power (*x*-axis) on the mean connectivity (degree, *y*-axis). The approximate scale-free topology can be attained at the soft-thresholding power of 8. **(B)** Gene clustering dendrograms of DEGs, with dissimilarity based on consensus topological overlap, together with assigned module colors indicated by the color row. Each colored row represents a color-coded module, which contains a group of highly connected genes. A total of 27 modules were identified. **(C)** Relationships of consensus module eigengenes and fungal biomass of *F. graminearum*. Each row in the table corresponds to a consensus module shown on the left side of each row. Numbers in the table indicated the correlations of the corresponding module eigengenes and fungal biomass trait, with the *p* values shown in parentheses. The table is color coded by correlation according to the color legend. Intensity and direction of correlations are indicated on the right side of the heatmap, in which red indicated positively correlated and green indicated negatively correlated. MTR, module–trait relationship.

To ascertain the functional gene co-expression networks involved in disease resistance to GSR, we deployed WGCNA to analyze the DEGs identified from all samples. Co-expression analysis of genes associated with BPs at different time points upon infection with *F. graminearum* was conducted with the soft threshold of β = 8 (*R*^2^ = 0.9) (Figure 4A). Twenty-seven modules were identified (Figures 4B,C), among which the eigengenes of seven modules (turquoise, blue, pink, green, black, dark turquoise, dark gray) were shown to be significantly correlated with *F. graminearum* biomass (*p* < 0.01, Figure 4C).

**FIGURE 5:**
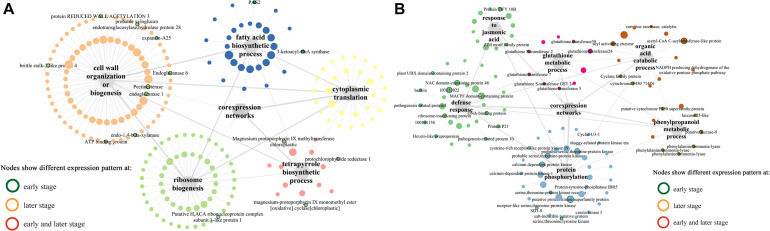
Co-expression networks of top 5 GO terms (biological process) constructed by WGCNA. **(A)** Turquoise module. **(B)** Blue module. The gray hexagons represent gene groups enriched in corresponding GO terms, and the gray lines linked with hexagons represent the connectivity between genes in corresponding GO term; the wider the line, the higher connectivity the genes. The node with colored border indicates different expression pattern at either early or later or both infection stages between susceptible and resistant lines.

Among the modules identified, the turquoise and blue modules are negatively and positively correlated with *F. graminearum* biomass, respectively. Based the cutoff criteria (| module membership| > 0.8 and | gene significance| > 0.2), genes with high connectivity in the significant module were identified. The co-expression network in turquoise module consisted of top five GOs enriched in BP (Figure 5A), including “ribosome biogenesis (GO:0042254),” “cytoplasmic translation (GO:0002181),” “cell wall organization or biogenesis (GO:0071554, including cellulose metabolic process and glucan metabolic process),” “fatty acid biosynthetic process (GO:0006633),” and “tetrapyrrole biosynthetic process (GO:0033014)” (Figure 5A). Moreover, the co-expression network from the top five BP GOs in blue module consisted of “defense response” (GO:0006952, including response to jasmonic acid), “organic acid catabolic process” (GO:0016054, including benzene-containing compound metabolic process), “glutathione metabolic process” (GO:0006749), “protein phosphorylation” (GO:0006468), and “phenylpropanoid metabolic process (GO:0009698)” (Figure 5B).

### Identification of Key Genes Differentially Expressed at Early and Later Stages in Response to Infection With *F. graminearum*

To identify the genes that specifically displayed differential expression pattern associated with distinct infection stage between susceptible and resistant lines upon infection, we conducted K-means clustering analysis using a series of genes termed in above GO categories (Supplementary Figure 6). Among the clusters with obviously different patterns at early stages (6, 12, and 24 hpi), cluster 5 showed strong up-regulation for genes in susceptible line, whereas down-regulation in resistant line; for cluster 7, no significant change was observed in susceptible line, yet dynamic changes in resistant line. Moreover, for cluster 15, genes were significantly down-regulated in susceptible samples, whereas up-regulation in resistant samples. For early stage cluster 24, susceptible and resistance samples show opposite pattern dynamic changes (Supplementary Figure 6A). Among the clusters with obviously different patterns at later stages (48 and 72 hpi), cluster 6 showed significant down-regulation in resistant line, whereas no changes were observed in susceptible samples; for cluster 8, susceptible and resistance samples showed opposite pattern with dynamic changes; however, for cluster 10, no significant change was found in susceptible line, whereas resistant samples displayed significant up-regulation (Supplementary Figure 6B). The distinctness of K-means clusters between susceptible and resistant lines at early and late stages were also analyzed by the ratio of relative standard deviation within susceptible/resistance and relative standard deviation in total (Supplementary Figures 6C,D).

**FIGURE 6:**
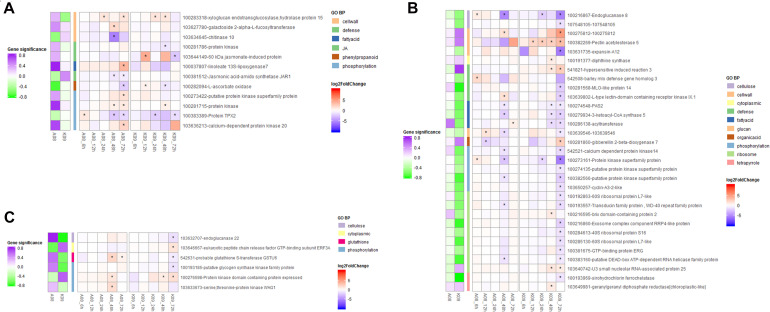
Heatmaps of expression levels of 49 genes enriched in top 5 biological process GO terms identified through K-means clustering. **(A)** Heatmap of genes with significance discriminative in A08 from K09 at different stages. **(B)** Heatmap of genes with significance discriminative in K09 from A08 at different stages. **(C)** Heatmap of genes with significance showing opposite pattern between K09 from A08 at different stages. The different colors in the heatmap represent log2 fold change of each gene at different time points labeled on the bottom of the map, and stars indicate the statistical significance at *p* < 0.05. The colored side squares on the left of heatmap represent the overall significance level of each gene corresponding to that in the heatmap. The side bars with different color represent different GO terms shown on the right.

To identify key genes genome-widely that might play important roles in immunity to GSR at early and later stages of maize in response to *F. graminearum*, respectively, we conducted K-means clustering analysis to select the genes with high connectivity displaying obviously different expression patterns between resistant and susceptible lines (Figure 6). In total, 407 genes displaying different expression patterns between two lines at both early and later stages were identified from K-means clustering. Those genes were significantly enriched in “defense response” (GO0006952), “response to jasmonic acid” (GO0009753), “phenylpropanoid metabolic process” (GO0009698), “glutathione metabolic process” (GO0006749), etc. (Supplementary Figure 6E), which was also in line with that identified in the co-expression networks by WGCNA. To identify the hub genes involved in the resistance at later stages of *F. graminearum* infection, we set | gene significance| > 0.2 as cutoff criteria and grouped the correlation of genes with *F. graminearum* biomass trait into three types, including negative correlation, no-significant correlation, and positive correlation; genes displaying different correlation groups between A08 and K09 were filtered. The connectivity of genes in A08 and K09 were then calculated with threshold value of 0.167 (0.8^8), and genes with no co-expression were removed; finally, 49 genes showing significantly different correlation between susceptible and resistant samples were filtered. Among those genes, 12 genes were either specifically discriminatory in A08 from K09 (Figure 6A), or discriminatory in K09 from A08 (Figure 6B), or displayed opposite pattern between two lines (Figure 6C). Therefore, the genes from these GO terms were selected and used as “pray genes” to identify their highly co-expressed top five hub genes. “Pray genes” and top five genes co-expressed with pray genes were determined and utilized to construct the co-expression networks in K09 and A08, respectively (Supplementary Figure 7A).

In the network constructed from turquoise module, only several *Fasciclin-like arabinogalactan protein* linked with three groups, suggesting their potential role in the network regulation of GO BP enriched, whereas “hub genes” in this module do not show different expression pattern between susceptible and resistance samples (Supplementary Figure 7A).

**FIGURE 7:**
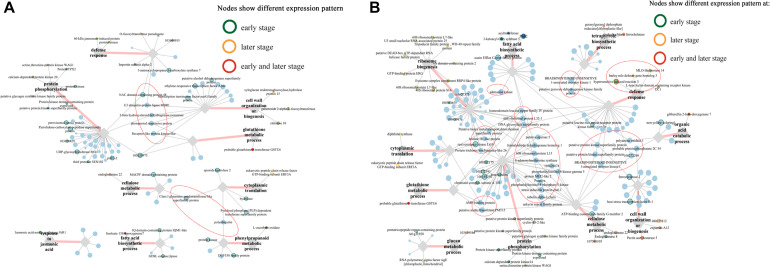
Co-expression networks of top 5 genes co-expressed with pray genes, specifically correlated with *F. graminearum* biomass in **(A)** A08 and **(B)** K09, constructed by WGCNA. The gray hexagons on the graph represent pray genes grouped by GO terms, and each node linked between hexagons represents genes in corresponding GO term; the wider the line, the higher connectivity the genes. The nodes linked with diamond represent genes co-expressed with pray genes grouped by GO terms. Genes in red dashed ellipse represent “hub genes” that co-expressed with more than one group of pray genes, and nodes with colored border indicate different expression patterns between two lines.

Intriguingly, multiple pray “hub genes” in the network constructed from blue module were evidently distinguished in their expression patterns between two lines at early stages (Figure 7). For example, *ethylene-responsive transcription factor ABI4* (103628663) linked with four clusters (“defense response,” “protein phosphorylation,” “glutathione metabolic process,” and “phenylpropanoid metabolic process”); three genes including *E3 ubiquitin-protein ligase ATL31* (103632881), *putative protein kinase superfamily protein* (100273687), and *putative carboxylesterase 15* (100274216) linked with three clusters, respectively; *putative RING zinc finger domain superfamily* was co-expressing with clusters “defense response” and “glutathione metabolic process,” whereas *putative transcription factor bHLH041* co-expressed with “defense response” and “protein phosphorylation” (Figure 7A). Furthermore, six genes including *WRKY transcription factor 6*, *BTB/POZ domain-contain protein, SKIP11*, *E3 ubiquitin-protein ligase RGLG1*, *receptor-like protein kinase-like*, and *GLTP family protein* linked with all six clusters (Figure 7A). In addition, in blue module network, two *laccase* genes (103640860, *laccase-25-like*; 103627746, *putative laccase-9*) and a number of *phenylamine ammonia-lyases* (PALs) (100281532, *phenylalanine ammonia-lyase*; 100381820, *phenylalanine ammonia-lyase*; 103627433, *phenylalanine ammonia-lyase*) in “phenylpropanoid metabolic process,” one *putative protein kinase superfamily protein* (100273687), and one *NAC domain-containing protein 48* (101027155) related to defense response were all co-expressing with cluster “protein phosphorylation” (Figure 7A).

In the network of A08, the susceptible line, only one gene at an early stage, *NAC domain containing protein 48* was found to co-express with genes in GO “defense response” and “protein phosphorylation,” whereas another gene “*class I amidotransferase-like superfamily protein*” co-expressed genes in GO “cellulase metabolic process” and “cytoplasmic translation” (Supplementary Figure 7B), suggesting these two genes might be associated with the susceptibility in A08. Furthermore, among those genes in the network of K09, four genes related and co-expressed with GO “defense response” were specifically correlated with *F. graminearum* biomass in K09 and showed strong co-expression with more than one group of pray genes, including *brassinosteroid insensitive 1-associated receptor kinase 1* (100281527) and *putative pyruvate dehydrogenase kinase family protein* (100193944) co-expressed with “defense response” (541821, *HIR3*) and “tetrapyrrole biosynthetic process” (100193969, *sirohydrochlorin ferrochelatase*); *putative protein kinase superfamily protein* (100383313) and *brassinosteroid insensitive 1-associated receptor kinase 1* (100274438) co-expressed with “defense response” (100281568, *MLO-like protein 14*) and “cellulose metabolic process” (103632707, *endoglucanase 22*) (Figure 7B), Thus, these genes in the co-expression networks might play positive important roles in GSR resistance.

### Identification of *HIR3-BAK1* Co-expression Network

**FIGURE 8:**
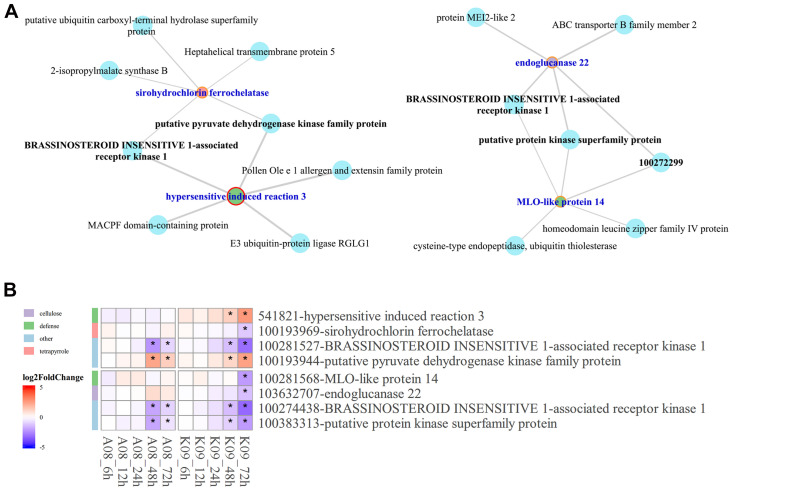
Identification of hub genes in the co-expression networks constructed by WGCNA. **(A)** The subnetworks reconstructed using the pray genes and co-expressed hub genes identified from WGCNA co-expression networks using genes enriched in top 5 GO terms. Key hub genes were highlighted in blue letter and red circle. **(B)** Heatmaps of pray genes and hub genes identified in co-expression networks showing their co-expression pattern. The side bars with different color represent different GO terms shown on the left. The different colors in the heatmap represent log2 fold change of each gene at different time points labeled on the bottom of the map, and stars indicate the statistical significance at *p* < 0.05.

Given that *BAK1* co-expressed significantly with several pray genes at the late stage upon *F. graminearum* infection, to understand the relationship between the co-expression of *HIR3* with *BAK1* and the resistance to GSR in K09, we reconstructed the subnetworks by pulling out the genes that were identified in large co-expression network. As shown in Figure 8A, strong co-expression was shown for *BAK1* with *HIR3* in GO term “defense response” and with *sirohydrochlorin ferrochelatase* in GO term “tetropyrrole biosynthetic process” in K09. Moreover, in another subnetwork, *BAK1* also showed strong co-expression with *MLO-like protein 14* and *endogluconase 22*, respectively (Figure 8A). The co-expression pattern of those genes in above subnetworks was visualized in the heatmap (Figure 8B), the majority of which displayed similar expression pattern as *BAK1*, mostly down-regulated, whereas *putative pyruvate dehydrogenase kinase family protein* was up-regulated significantly at the late stage (48 and 72 hpi), in both A08 and K09. Intriguingly, HIR3 was only up-regulated significantly in K09 at 48 and 72 hpi (Figure 8B), suggesting that it might be positively associated with the resistance to GSR.

### Metabolites Profiling in Resistant and Susceptible Lines Upon Infection With *F. graminearum*

**FIGURE 9:**
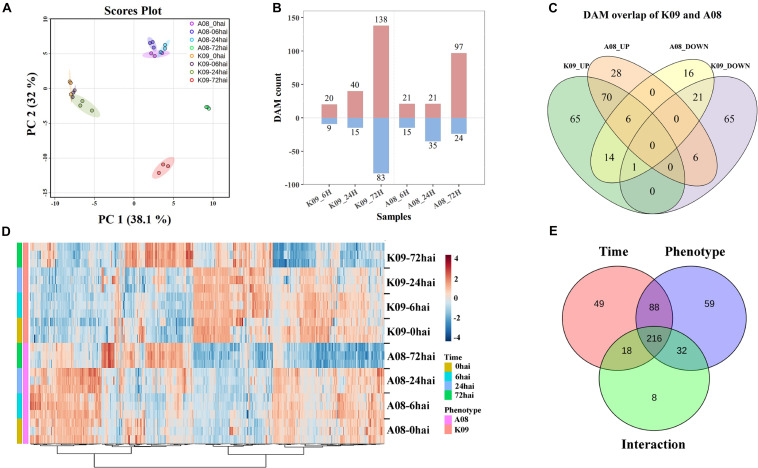
Metabolomic analysis of K09 and A08 stems at different time points upon infection with *F. graminearum*. **(A)** PCA score plot based on the metabolite profiling of K09 and A08. **(B)** The total number of up- and down-regulated differentially accumulated metabolites (DAMs) identified at different time points in K09 and A08. **(C)** Venn diagram illustrating the unique and common DAMs between K09 and A08. **(D)** Heatmap of DAMs identified to be significantly discriminatory between K09 and A08 upon infection with *F. graminearum*. The side bars on the left of heatmap represent genotypes and time points, respectively. The statistical significance was analyzed by two-way ANOVA (interaction *p* ≤ 0.01). **(E)** The summary of the number of DAMs discriminated by time points, phenotypes, and interaction between time points and phenotypes.

To determine the metabolites highly associated with the resistance to GSR, we deployed a suite of UPLC-MS/MS methods to perform a global metabolic profiling in A08 and K09 postinfection with *F. graminearum*. The combined analysis was conducted in three biological replicates, each containing the designated stem samples collected from 10 individual seedling at three time points, 6, 24, and 72 hpi, with non-infected materials as controls, respectively. PCA plot showed that while the biological replicates were closely grouped in the space, supporting the good correlation between replicates, the obvious separation between two lines was observed by PC1 and that of time points by PC2, with a clear distance of the time point 72 hpi from others (Figure 9A), indicating the strong and specific activation of metabolites at a later stage in both lines.

The one-way ANOVA for each time point revealed the substantial changes (*P* < 0.05) in the levels of numerous metabolites within different treatments. In total, 518 DAMs were determined from all treatments, including 305 from K09 and 213 from A08, respectively (Figure 9B). Among those DAMs, there were 20 and 40 up-regulated, 9 and 15 DAMs down-regulated metabolites in K09 at 6 and 24 hpi, respectively. However, more DAMs, including 138 and 83 up-regulated and down-regulated, respectively, were identified at 72 hpi in K09. Similarly, 21 each up-regulated and 15 and 35 down-regulated DAMs at 6 and 24 hpi, whereas more DAMs, e.g., 97 up-regulated and 24 down-regulated, were detected at 72 hpi in A08, respectively (Figure 9B). The different numbers of DAMs clearly indicated the highly distinctive pattern of metabolites between two different stages, e.g., early stage (6 and 24 hpi) versus late stage (72 hpi), supporting the gene expression patterns identified by RNA-seq. Furthermore, the numbers of common and specific DAMs were highlighted in a Venn diagram between two lines. There were 28 and 65 metabolites significantly increased, whereas 16 and 65 metabolites were significantly decreased in A08 and K09 in response to *F. graminearum* infection, respectively (Figure 9C). Moreover, 70 and 21 metabolites were detected to be commonly increased and decreased between two lines, and 27 compounds showed opposite patterns in resistant and susceptible lines, respectively (Figure 9C).

To explore the globally discriminatory metabolites significantly altered between K09 and A08, the heatmap with significant features was constructed by time-series analysis (two-way ANOVA) between A08 and K09 at different time points upon infection. These clusters with highly correlated metabolites clearly illustrated the distinct differences of DAMs between two lines; e.g., the majority of DAMs increased in A08 were decreased in K09, and *vice versa* (Figure 9D). With the interaction between time and phenotype, the dynamic changes of 216 metabolites were identified to be significantly different between A08 and K09 across all infection stages (Figure 9E). Through KEGG metabolic pathway enrichment, the discriminative metabolites in K09 in response to *F. graminearum* infection were mainly involved in anthocyanin biosynthesis, ubiquinone, and other terpenoid–quinone biosynthesis, pyrimidine metabolism, biosynthesis of secondary metabolites, and others (Supplementary Figure 8A), whereas those in A08 were mainly associated with isoflavonoid biosynthesis, flavonoid biosynthesis, purine metabolites, and others (Supplementary Figure 8B).

**FIGURE 10:**
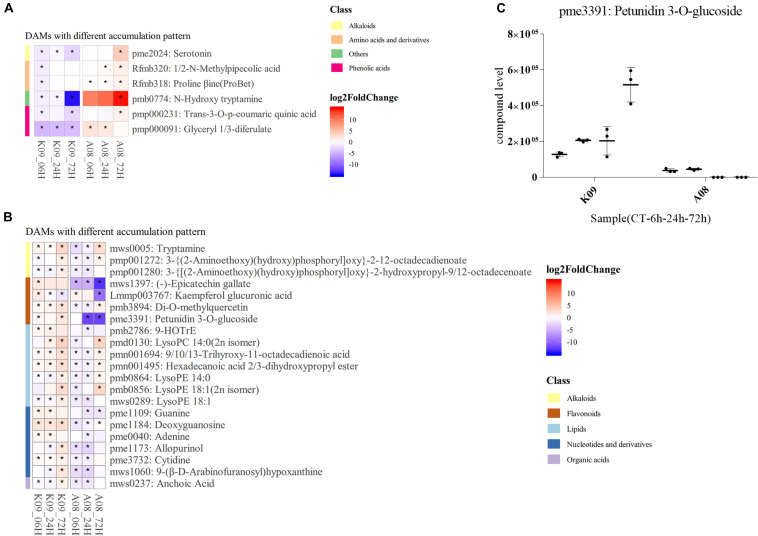
Identification of significantly discriminative metabolites between K09 and A08 upon infection with *F. graminearum*. **(A)** DAMs up-regulated in K09, but down-regulated in A08. **(B)** DAMs down-regulated in K09 but up-regulated in A08. **(C)** The quantitative comparison of petunidin 3-*O*-glucoside (pme3391) contents in the stems of K09 and A08 at different time points upon infection with *F. graminearum*. The side bars with different color on the left of heatmap of **(A)** and **(B)** represent different classes of metabolites shown on the right. The different colors in the heatmap represent log2 fold change of each metabolite at different time points between two lines labeled on the bottom of the map, and stars indicate the statistical significance by two-way ANOVA (*p* ≤ 0.01).

According to KEGG enrichment of DAMs differed between two lines, several compounds were found to be significantly differentially accumulated in each line, respectively. Among those, serotonin (pme2024) and *N*-hydroxyl tryptamine (HTAM) (pmb0774) formed from tryptamine (TAM) by the flavin monooxygenase–like protein (YUCCA), which is a key enzyme in auxin metabolism, were highly accumulated in A08, especially at 72 hpi (Figure 10A); glyceryl 1,3-diferulate (pmp000091) was mainly increased at early time points, whereas *N*-methylpipecolic acid (Rfmb320), proline betaine (Rfmb318), and *trans*-3-*O*-*p*-coumaric quinic acid (pmp000231) were found to be accumulated at 72 hpi (Figure 10A). Numerous metabolites mainly from alkaloids, flavonoids, lipids, nucleotides, and derivatives, clusters, and organic acids, were accumulated higher in K09 than in A08 (Figure 10B), among which the polyphenol epicatechin gallate (EGCG) (mws1397), Kaempferol glucuronic acid (Lmmp003767), and petunidin 3-*O*-glucoside (pme3391), belonging to flavonoids, were significantly accumulated in K09 than in A08, particularly at the late stage (72 hpi). Multiple DAMs from lipids metabolism, including 9-HOTrE (pmb2786), 9/10/13-trihydroxy-octadecadienoic acid (pmn001694), hexadecanoic acid 2/3-dihydroxypropyl ester (pmn001495), LysoPC (14:0, 2n isomer) (pmd0130), and several derivatives of LysoPE (14:0, pmb0864; 18:1, 2n isomer, pmb0856; 18:1, mws0289), also showed significantly higher contents in K09 than A08, especially at an early time (6 and 24 hpi) (Figure 10B). Furthermore, the quantitative analysis of petunidin 3-*O*-glucoside showed that it was accumulated to greater levels in K09 at each time point than that in A08 (Figure 10C).

### *zmhir3* Mutant Is More Susceptible to GSR

As the potential involvement of petunidin 3-*O*-glucoside in the resistance to GSR, we conducted a joint analysis of metabolites with RNA-seq data, by evaluating the top 20 genes that showed strong correlation with petunidin 3-*O*-glucoside (Pearson correlation > 0.9). Among those genes, *polyadenylate binding protein RBP47* (Zm00001d0505838) and *inositol monophosphatase 3* (Zm0000103110) showed the most significantly negative (−0.955) and positive (0.954) correlations, respectively (Supplementary Table 4). In addition, *HIR3* also showed the higher positive correlation with petunidin 3-*O*-glucoside (Pearson correlation = 0.936), which had a strong linear correlation (*R*^2^ = 0.874) for RNA-seq reads of *HIR3* and metabolite counts of petunidin 3-*O*-glucoside (pme3391) (Supplementary Figure 9A). Moreover, *HIR3* RNA-seq reads, *FH* RNA-seq reads, and pme3391 counts showed a similar pattern, which were increasing steadily along with the time points in resistant line K09, whereas very low levels for all three in susceptible line A08 (Supplementary Figure 9B), supporting the involvement of *HIR3* in maize resistance to GSR.

**FIGURE 11:**
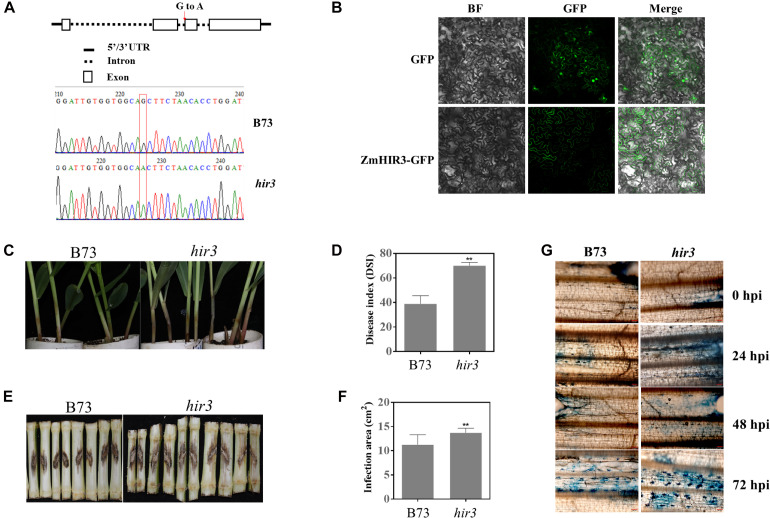
Identification and GSR phenotypic analysis of *zmhir3* mutant in response to *F. graminearum* infection. **(A)** Identification of *zmhir3* EMS mutant by resequencing. The top schematic diagram represents the SNP mutation in *ZmHIR3* gene. The red arrow shows the mutation of G to A in the end of second intron of gene. The bottom indicates the mutation of SNP G to A by resequencing. **(B)** Subcellular localization of *ZmHIR3* on the plasma membrane. BF, bright field. **(C)** Seedling GSR phenotypes of *zmhir3* mutant versus wild-type B73. The phenotype was recorded at 3 dpi. **(D)** Quantification of seedling GSR disease index (DSI) of *zmhir3* mutant vs. wild-type B73. **(E)** Field GSR phenotypes of *zmhir3* mutant vs. wild-type B73 recorded at 15 dpi. **(F)** GSR disease areas on the stems of *zmhir3* mutant vs. wild-type B73 adult plants in the field condition. ^∗∗^Statistically significant (*p* < 0.01) between mock and inoculation treatments analyzed by *t*-test. **(G)** Trypan blue staining of cell death on the stems of *zmhir3* mutant vs. B73 seedlings at different time points upon infection with *F. graminearum*. The scale on the picture is 50 μm. hpi, hours postinfection.

Given the potential involvement of HIRs and BAK1 in the regulation of cell death, the finding that *HIR3* and *BAK1* are highly co-expressed in same network prompted us to examine whether HIR3 is indeed involved in GSR resistance through the regulation of cell death. We obtained the mutant *zmhir3* from a maize EMS mutant library (Lu et al., 2017) and confirmed the point mutation in the mutant by sequencing (Figure 11A). The mutation occurred on the boundary of second intron and third exons of *ZmHIR3*, where the last “G” of right “AG” in intron was mutated to “A,” which resulted in the extension of the second intron flanking the third exon (Figure 11A). To examine the subcellular localization of ZmHIR3, we constructed ZmHIR3-GFP and overexpressed it transiently in *N. benthamiana* leaves, using GFP alone as a control. While GFP was found to be distributed at the plasma membrane, cytosolic, and nucleus, ZmHIR3-GFP signal was only detected at the plasma membrane (Figure 11B), confirming ZmHIR3 is a plasma membrane-localized protein. Furthermore, the seedling assay upon infection with *F. graminearum* showed that *zmhir3* mutant was more susceptible to this pathogen, compared to WT B73 (Figure 11C), evidenced by significantly greater disease index in the mutant compared to WT (Figure 11D). To confirm the phenotype, we examined the GSR phenotype of adult plants of *zmhir3* and WT in the field condition. In line with seedling phenotypes, *zmhir3* mutants displayed significantly greater lesion size, compared to WT (Figures 11E,F). Trypan blue staining using the stems upon infection with *F. graminearum* showed stronger cell death in *zmhir3* mutant than WT at each time points, especially at 72 hpi, supporting that the more obvious necrotrophic phase of the mutant was likely associated with the susceptibility to *F. graminearum* (Figure 11G).

To better understand how *HIR3* is involved in the regulation of resistance to GSR, we sought to investigate the expression levels of several representative genes involved in disease resistance or immunity to pathogens. While *HPL* and *AOC1*, which are related to JA pathway, showed strong induction in *zmhir3* mutant, but not WT, at 6 hpi; however, both were induced to higher levels in WT, but suppressed to the similar level as control in *zmhir3* mutant at 24 hpi (Supplementary Figure 10). Similar expression pattern was observed for *MYC7* at 24 hpi, which was induced to higher level in both WT and mutant at 6 hpi. *EDS1*, which is a key gene associated with SA signaling pathway, and *MPK3* in PTI pathway, were not activated at 6 hpi in both lines, whereas activated in WT but suppressed in *zmhir3* mutant at 24 hpi (Supplementary Figure 10). Taken together, these data suggested that JA-biosynthetic pathway genes were activated earlier in *zmhir3* mutant compared to WT, whereas those genes as well as that in SA pathway and PTI pathway are likely associated with later activation during maize interaction with *F. graminearum*.

## Discussion

### Multiple Gene Co-expression Networks Associated With Immunity to *F. graminearum*

Previously, numerous works have attempted to dissect the complex molecular mechanisms of maize–*Fusarium* interactions through discovering key genes using transcriptomic and metabolomic approaches, as well as the functional studies. For example, Ye et al. (2018) deployed the combination of transcriptome and cytological approaches using a pair of resistant and susceptible near-isogenic lines (NILs) and identified a series of genes associated with lignins, phenolic acids, secondary metabolites, and phytohormone pathways that might play important roles in the resistance to GSR (Ye et al., 2013). Furthermore, a comparative transcriptomic analysis using three NILs, e.g., NIL1 (with *qRfg1*), NIL2 (with *qRfg2*), and NIL3 (with neither *qRfg1* nor *qRfg2*), demonstrated the essential role of *qRfg1* in GSR resistance through constitutively induced expression of defense genes. Moreover, using the comparative metabolomics, two maize metabolites, smilaside A and smiglaside C, were identified in maize seeding roots in response to *F. graminearum*, the relative abundance of which was regulated by ethylene signaling, thereby to activate the immunity to this pathogen (Zhou S. et al., 2019). Despite the progress, the complex regulation networks of host genes associated with GSR resistance, especially the evidence underlying the activation of cell death response at distinct infection stages of *F. graminearum*, were not extensively investigated.

To explore the complex mechanism underlying the regulation of distinct immunity responses during differential infection stage of *F. graminearum*, we deployed a combination of transcriptomics and metabolomics using a pair of inbred lines with contrast disease phenotypes to dissect the genes associated with maize GSR resistance. Through WGCNA analysis (Langfelder and Horvath, 2008), multiple key gene co-expression networks centered by hub genes were identified. For example, the co-expression network in blue module mainly included “defense response,” “organic acid catabolic process,” “glutathione metabolic process,” “protein phosphorylation,” and “phenylpropanoid metabolic process” (Figure 5). In the network of “defense response,” multiple genes showed differential expression pattern between two infection stages, including *NAC domain containing protein 48*, *MACPF domain-containing protein*, *plant U-box containing protein 2*, *pathogenesis-related protein 5*, and *pathogenesis-related protein 10*. Additionally, two jasmonates signaling components, *protein TIFY 10B* and *ZIM motif protein family*, both belonging to JAZ family (Ma et al., 2021), were also found to be differentially expression at the early stage of *F. graminearum* infection. Although numerous studies have examined the function of jasmonates signaling components, including JAZs in model plant species, very little is known about their roles in maize–*Fusarium* interactions. Recently, we found that while exogenous methyljasmonates could enhance maize resistance to GSR, endogenous JA might function as a susceptibility factor, whereas the transcription repressor ZmJAZ15 played a positive role in GSR resistance (Ma et al., 2021). Considering the infection style-dependent expression pattern of JA signaling components identified in this study, it will be worthy to investigate more extensively in the future how those genes function in maize interaction with *F. graminearum* at distinct biotrophic and necrotrophic stages.

Besides the co-expression network “defense response,” “phenylpropanoid metabolic process” was also found to be likely associated with GSR resistance. Among this network, two *laccase* genes and three PALs were expressing differentially at the early stage of *F. graminearum* infection. It has been broadly reported that phenylpropanoid pathway is involved in the plant defense response against abiotic and biotic stresses, mainly through activating the biosynthesis of secondary metabolic compounds, such as flavonoids, lignins, hydroxycinnamic acid, coumarins, and stilbenes (Yu and Jez, 2008; An and Mou, 2011; You et al., 2020), whereas PAL is believed to be responsible for catalyzing the essential step of salicylate biosynthesis (Dixon and Paiva, 1995; Lee et al., 1995). In maize, PAL genes were reported to be involved in the resistance against nematodes (Starr et al., 2014) and sugarcane mosaic virus (SCMV) infection likely through activation of SA biosynthesis (Yuan et al., 2019). It awaits to be investigated in the future whether and how the components of phenylpropanoid pathway, particularly PAL and SA, are differentially involved in maize resistance against GSR during distinct biotrophic and necrotrophic stages upon *F. graminearum* infection.

Another issue that one might be concerned with is the interaction of mechanic wounding with cell death response during *F. graminearum* infection. In this study, while massive DEGs were identified at later stages of infection (48 and 72 hpi), much smaller number of genes in response to wounding were identified to be DEGs compared to that associated with defense response (Figure 3A). Moreover, we could not detect significant DEGs commonly overlapped between wounding-inducible and cell death–associated processes, indicating much less interference of cell death–associated genes by wounding. Previous studies showed that wounding-inducible genes in maize were usually influenced in the first few hours, which could more likely mimic at certain level the effect of insect feeding (Tzin et al., 2015; Wang et al., 2017b). However, further comprehensive study should be considered in the future to investigate extensively how wounding signaling would interfere the defense response to fungal pathogens.

### Potential Involvement of Anthocyanin in Maize Resistance to GSR

Anthocyanins belong to the family of flavonoids identified in the most plant species. In addition to SA biosynthesis pathway, the intermediate product of PAL-catalyzed pathway, cinnamic acid, is also metabolized to *p*-hydroxyl-cinnamic acid and then formed to tetrahydroxychalcone via chalcone synthase (CHS)–catalyzed condensation of *p*-coumaroyl-coenzyme A (COA) and three molecules of malonyl-COA (Dixon and Paiva, 1995; Holton and Cornish, 1995). The tetrahydroxychalcone is further converted to naringenin chalcone and then to naringenin, which is the metabolized to dihydroxy-kaemopferol, the direct precursor of other flavones, flavanones, flavanols, and anthocyanins, through either F3H (flavonoid-3′-hydroxylase) or DFR (dihydroflavovol-4-reductases) (Holton and Cornish, 1995).

Anthocyanins have been known to play roles in defense response against diverse pathogens. For instance, inoculation with the fungus *Cochliobolus heterostrophus* on sorghum resulted in the dramatic reduction of the light-induced accumulation of anthocyanin, which could be exerted by repressing the expression of the anthocyanin biosynthesis genes *F3H*, *DFR*, and *anthocyanidin synthase*, whereas the activation of *PR10*, *PAL*, and *CHS*, as well as the synthesis of 3-deoxyanthocyanidin phytoalexins (Lo and Nicholson, 1998). Moreover, overexpressing maize C2 (colored-2, encoding chalcone synthase) gene in rice could enhance the resistance to blast fungus *Magnaporthe grisea* (Gandikota et al., 2001). Another study in maize showed that the effector protein TIN2 of maize fungal pathogen *Ustilago maydis* could target host protein kinase ZmTTK1, which is involved in the regulation of anthocyanin biosynthesis, to modulate anthocyanin, as well as lignin pathways, thereby to facilitate the pathogenicity of *U. maydis* (Tanaka et al., 2014). However, there was no report about the role of anthocyanin in maize against GSR.

Through metabolomics and association analysis, we found that several flavonoid compounds were highly enriched in K09 than in A08 at the late stage of fungal infection, among which petunidin 3-*O*-glucoside (pme3391) was one of the most accumulated DAMs in resistant line upon fungal infection. Petunidin 3-*O*-glucoside is one type of anthocyanins, mostly found in fruits, berries, and red grapes (Baldi et al., 1995); however, very little is known about its involvement in disease resistance to plant pathogens. Given that petunidin 3-*O*-glucoside was significantly co-expressed with several key genes, especially HIR3, which is likely associated with defense response, it will be exciting to investigate more extensively the function of petunidin 3-*O*-glucoside in GSR resistance.

### Core Genes Displayed Infection Stage-Dependent Pattern in Response to *F. graminearum*

It has been known that *F. graminearum* possesses hemibiotrophic lifestyle upon the infection on host plants, such as wheat head and maize stalks. To encounter this hemibiotroph pathogen, plants have evolved complex defense strategies at both transcriptional and translational levels (Kazan and Gardiner, 2018). Previous studies have shown that multiple plant defense signaling pathways displayed spatiotemporal dynamics ad stage-specific pattern in response to pathogen attack (Ding et al., 2011; Zhang X.W. et al., 2012; Brown et al., 2017). For instance, SA and Ca^2+^ signaling pathways were activated at much earlier time, whereas JA signaling pathway was induced at the late stage of infection (Ding et al., 2011). Moreover, metabolites profiling on infected wheat coleoptiles suggested that *F. graminearum* takes advantage of primary metabolisms at its initial infection stage (< 24 hpi), whereas utilizes secondary metabolites for its spreading and virulence at a later stage, e.g., 64 hpi (Zhang X.W. et al., 2012). In maize–*C. graminearum* interaction, the fungus was able to overcome the host immunity by deploying differential strategies during initial biotrophic and later necrotrophic stages (Vargas et al., 2012). In this study, the combined transcriptome and metabolome analysis clearly showed the different patterns of DEGs and DAMs identified between two phases postinfection further supported that the distinctness and specificity of maize immune response to *F. graminearum* are strongly associated with the infection style of this fungus.

To identify hub genes associated with GSR resistance, we deployed WGCNA to construct co-expression networks, in which a series of genes were identified to be significantly enriched at the early stage of fungal infection, including *ethylene-responsive transcription factor ABI4*, *E3 ubiquitin-protein ligase ATL31*, *putative protein kinase superfamily protein*, *putative carboxylesterase 15*, and *putative RING zinc finger domain superfamily*, suggesting their potential involvement in defense response at the early stage upon infection. Furthermore, *NAC domain containing protein 48* and *class I amidotransferase-like superfamily protein* seemed to be associated with the susceptibility in A08 at a later stage, whereas *BAK1* co-expressed with *HIR3* and *MLO-like protein 14*, as well as *endoglucanase 22* associated with GO term “defense response,” suggesting that these genes might be directly involved in the resistance at a later stage to *F. graminearum*. The future work will be centered on dissecting the function and molecular mechanisms, including the signaling networks among these components, underlying the disease resistance regulated by these genes.

### The Role of HIR3 in Cell Death Regulation During Maize Interaction With *F. graminearum*

It has been well documented that cell death is highly associated with plant immunity to pathogens (Mukhtar et al., 2016; Huysmans et al., 2017; Eugenia et al., 2020). Cell death is usually considered as a ubiquitinous feature of plant when challenged with harsh environmental stress, which is tightly controlled by a series of signaling networks. Intriguingly, among the networks identified in this study, several subnetworks related to cell death were found to be highly correlated with the differential response to *F. graminearum* infection between two lines. In particular, ZmHIR3 seems to execute its role as a hub gene in the complex network of cell death regulation, particularly at necrotrophic stage of maize upon infection with *F. graminearum*.

Hypersensitive induced reaction 3 belongs to a superfamily of PID (proliferation, ion, and death), which is also considered as the member of stomatin/prohibitin/flotillin/HflK/C (SPFH) domain family (Lv et al., 2017). Multiple HIR genes have been identified in plant defense responses, such as TaHIR1 and TaHIR3 in wheat resistance to stripe rust (Duan et al., 2013), whereas four barley HIR genes were found to be associated with activation of HR response (Rostoks et al., 2003). Moreover, *Arabidopsis* HIR2 was associated with FLS2 in a *N. benthamiana* transient expression system (Qi and Katagiri, 2012), indicating the essential role of HIR protein in regulating PTI response. Using HPB tag, a number of potential components were identified as RPS2-interacting proteins, among which two HIR proteins were also pulled out (Qi and Katagiri, 2009), whereas another study using Forster resonance energy transfer (FRET) and pull-down assay found that AtHIR1 and AtHIR2 are physically associated with RPS2, and *Athir2-1* and *Athir3-1* mutants are susceptible to Pst DC3000 AvrRpt2 but not Pst DC3000, strongly suggesting the involvement of HIRs in ETI signaling pathway (Qi et al., 2011). Furthermore, using fluorescence correlation spectroscopy, fluorescence cross-correlation spectroscopy, fluorescence lifetime imaging, and FRET techniques, *Arabidopsis* AtHIR1 was proven to be colocalized with microdomain maker protein Remorin 1.4 (REM1.3) in microdomain of plasma membrane (Lv et al., 2017).

In maize, three HIR genes have been reported to be associated with defense response, including *ZmHIR1*, *ZmHIR2*, and *ZmHIR3*, among which the expression of *ZmHIR3* was found to be up-regulated in a lesion mimic mutant *Ler9*, suggesting its direct role in the cell death control (Nadimpalli et al., 2000). Notwithstanding these significant efforts, it remains unclear how maize HIR proteins function in the immunity to *Fusarium* infection.

In this study, we identified *ZmHIR3* as a hub gene in the co-expression network in the resistant line, and *zmhir3* mutant became more susceptible to GSR. Furthermore, the histological staining showed that *zmhir3* mutant accumulated more cell death compared to WT, especially at a later stage upon *F. graminearum* infection, suggesting that the cell death at necrotrophic phase in the mutant caused by fungal infection is highly related to the susceptibility; thus, ZmHIR3 is likely associated with GSR resistance. Moreover, we also found that ZmHIR3 not only co-expressed with *BAK1*, but also several other genes that have been known to be associated with cell death control, such as *MLO-like protein 14* and *endoglucanase 22.* BAK1 is a coreceptor of PRR localized at plasma membrane for perception of PAMPs to activate PTI and has been largely reported to play important roles in the regulation of cell death, involving not only the development-related cell death, but also defense-related cell death process (Gao et al., 2018). *MLO* is a susceptibility gene identified during barley interaction with powdery mildew (Jorgensen, 1992; Büschges et al., 1997) and has been known to be associated with the regulation of cell death in diverse plant species (Büschges et al., 1997; Piffanelli et al., 2002; Stein and Somerville, 2002). However, it remains unclear how ZmHIR3 cooperates with other components to regulate cell death during the interaction with *F. graminearum*. Together, despite our fragmentary evidence of ZmHIR3 in the resistance to GSR, through RNA-seq, metabolomics, and genetic experimentation, uncovering the potential involvement of ZmHIR3 in GSR resistance will be of utmost importance to enhance our understanding the role of cell death in mediating defense response against differential infection style of specific pathogens, especially the hemibiotrophic fungus *F. graminearum*.

Intriguingly, through joint analysis using RNA-seq and metabolomics, we found that HIR3 was highly correlated with anthocyanin petunidin 3-*O*-glucoside (pme3391) at a later stage of *F. graminearum* infection in resistant line K09, revealing the possibility of HIR3 and anthocyanin network in the regulation of GSR resistance. A recent study showed that apple HIR protein MdHIR4 could suppress anthocyanin accumulation via interacting with MdJAZ2 protein (Chen et al., 2017). It will be interesting to investigate whether *HIR3* gene regulates anthocyanin accumulation, or anthocyanin impacts *HIR3* expression, upon *F. graminearum* infection.

## Data Availability Statement

The original contributions presented in the study are included in the article/Supplementary Material, further inquiries can be directed to the corresponding author/s. The data presented in the study are deposited in the NCBI GEO database (accession number: GSE171968).

## Author Contributions

XG and YS did the conceptualization and experimental design. YS, XR, and QW did the data curation and validation. YS, XR, QW, and XG did the formal analysis. XG did the funding acquisition, project administration, and supervision. YS, XR, QW, YZ, LM, and FW did the investigation. YS, XR, QW, and XG did the methodology. YZ, ZW, and XG did the resources. XG, YS, and XR wrote – original draft preparation. XG, YS, XR, and QW wrote – review and edited. All authors read and approved the final manuscript.

## Conflict of Interest

The authors declare that the research was conducted in the absence of any commercial or financial relationships that could be construed as a potential conflict of interest.

## Footnotes

1 bioconductor.org

2 http://www.anpel.com.cn/

3 www.shimadzu.com.cn/

4 www.appliedbiosystems.com.cn/

5 www.r-project.org

6 http://www.kegg.jp/kegg/compound/

7 http://www.kegg.jp/kegg/pathway.html
