# Supplementary material for: Integrated Gene Co-expression Analysis and Metabolites Profiling Highlight the Important Role of ZmHIR3 in Maize Resistance to Gibberella Stalk Rot
Source: Front Plant Sci. 2021 May 11;12:664733. doi: 10.3389/fpls.2021.664733 (PMC8144520; doi:10.3389/fpls.2021.664733)
Supplement: Supplementary file 1 [file Data_Sheet_1.docx]

**
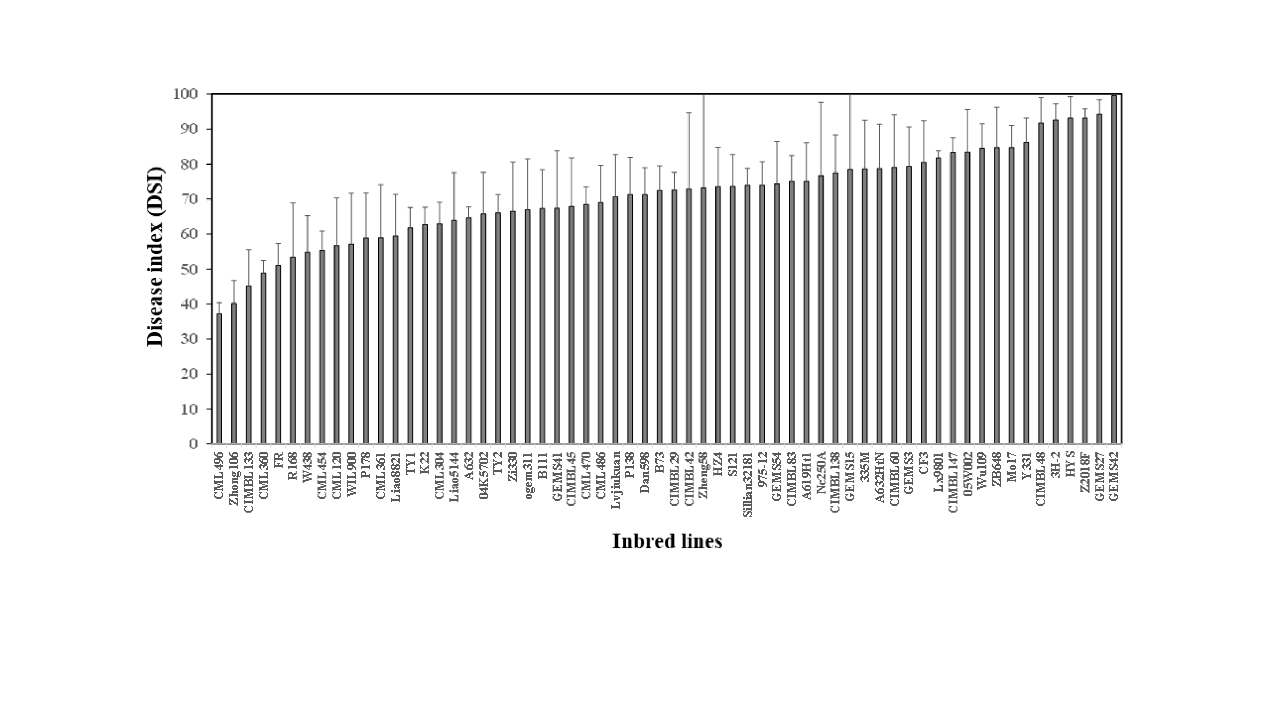
**

**Supplemental Figure S1.** Seedling phenotypes of GSR in 62 inbred lines. The phenotypes of *GSR* on two-week-old seedlings were scored at 3 days post inoculation (dpi) with *F. graminearum*. The disease index was calculated based on the phenotype data collected from at least 10 seedlings per line. The experiments were conducted at least three times, with at least ten seedings per line, and the data are shown as the means±SD of three replicates**.**


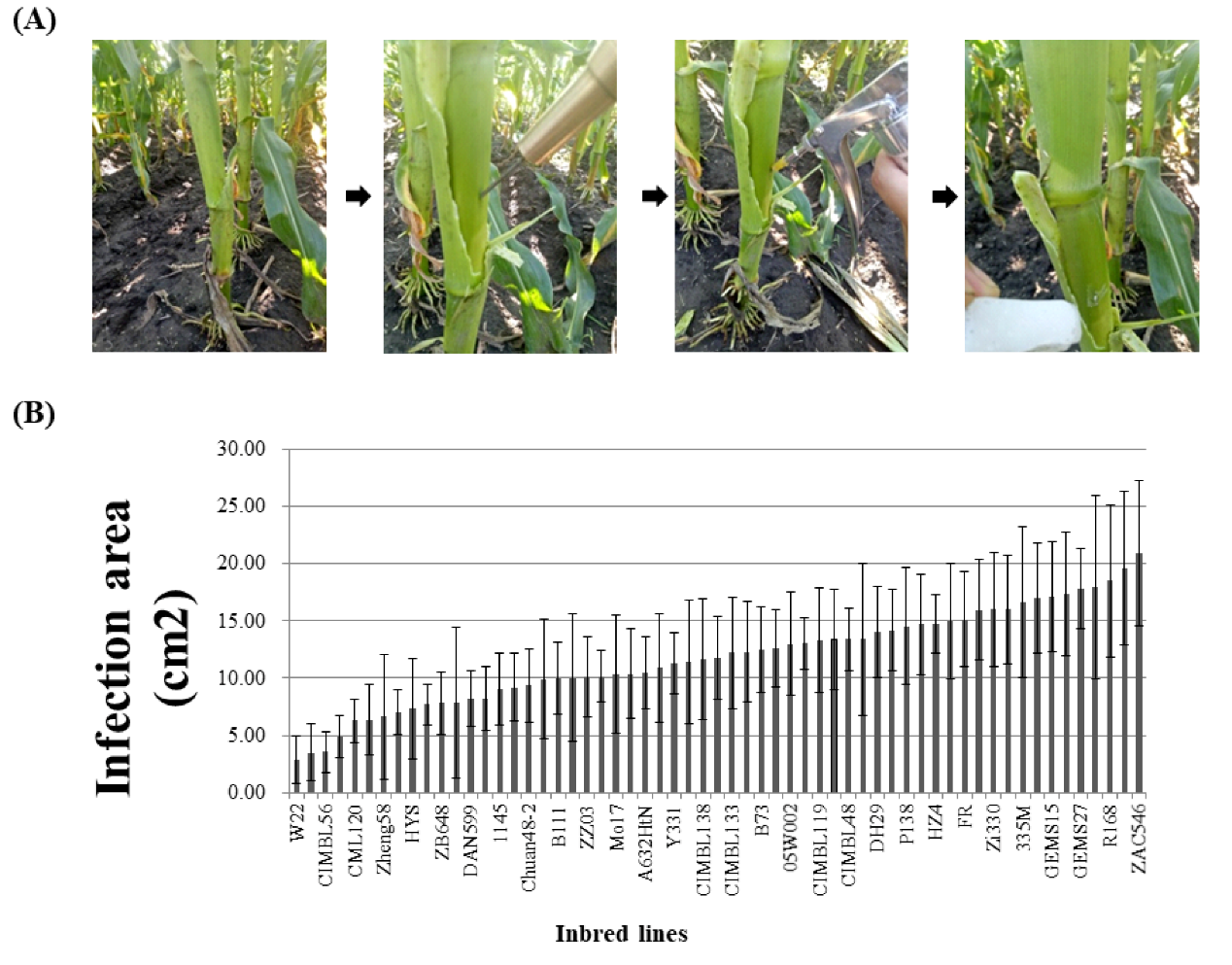


**Supplemental Figure S2.** Field GSR phenotypes in partial lines used. (**A**) Schematic diagram of GSR phenotyping method in the field condition. (**B**) Quantification of GSR phenotype different inbred lines in field assay. The disease area was measured at 15 dpi upon infection with *F. graminearum*.


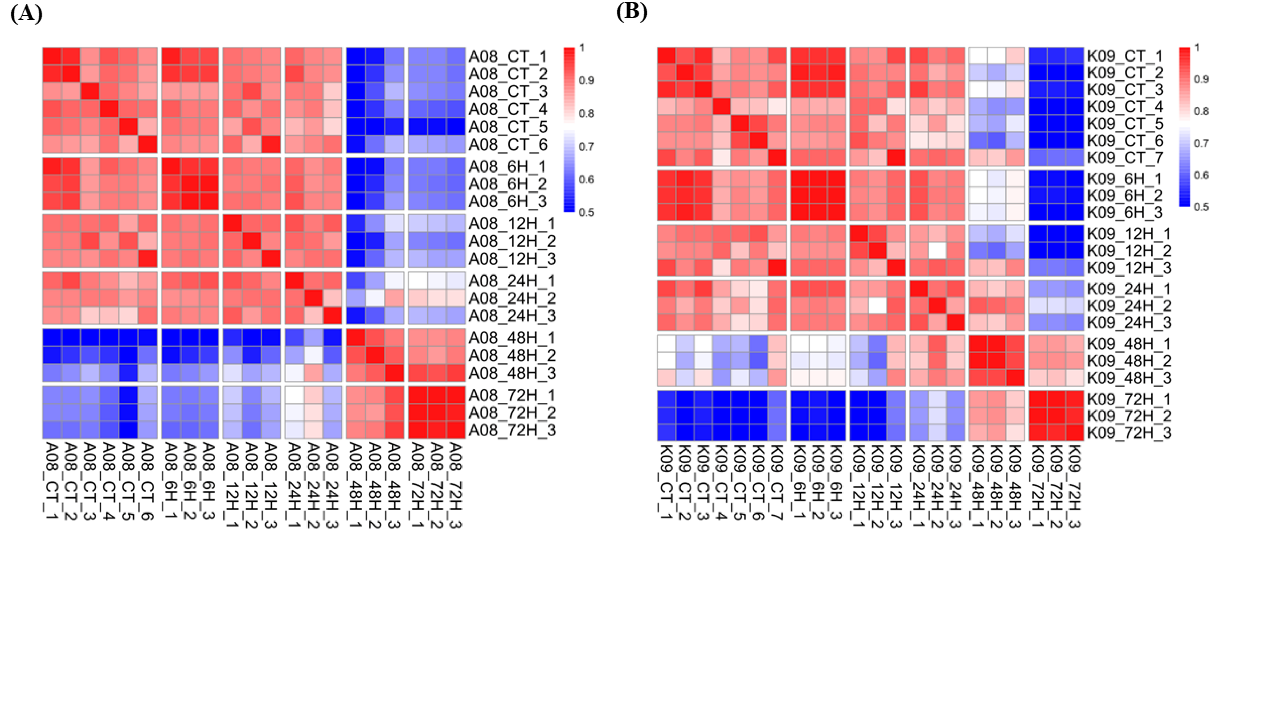


**Supplemental Figure S3.** Pearson correlation co-efficiency of RNA-seq among the samples collected at different time points upon infection with *F. graminearum*.in (**A**) A08 and (**B**) K09.


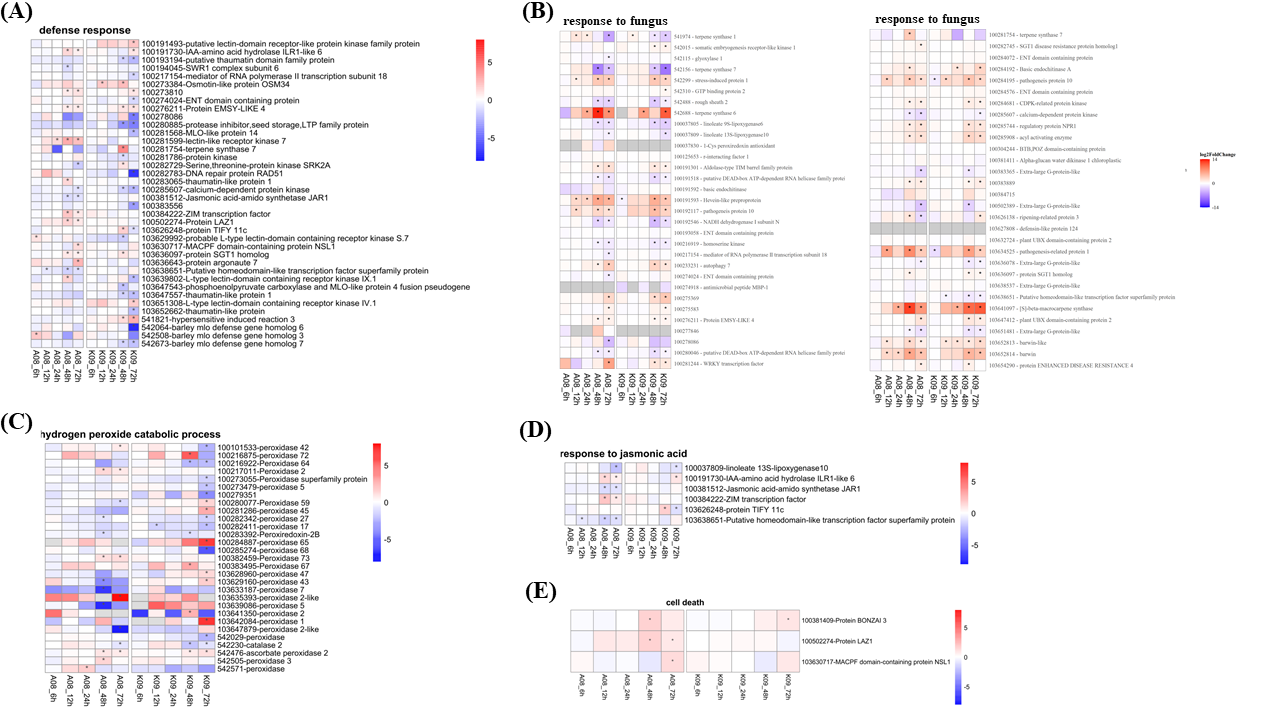


**Supplemental Figure S4.** Heatmap of DEGs involved in (**A**) defense response, (**B**) response to fungus, (**C**) hydrogen peroxide catabolic process, (**D**) response to jasmonic acid and (**E**) cell death.

**
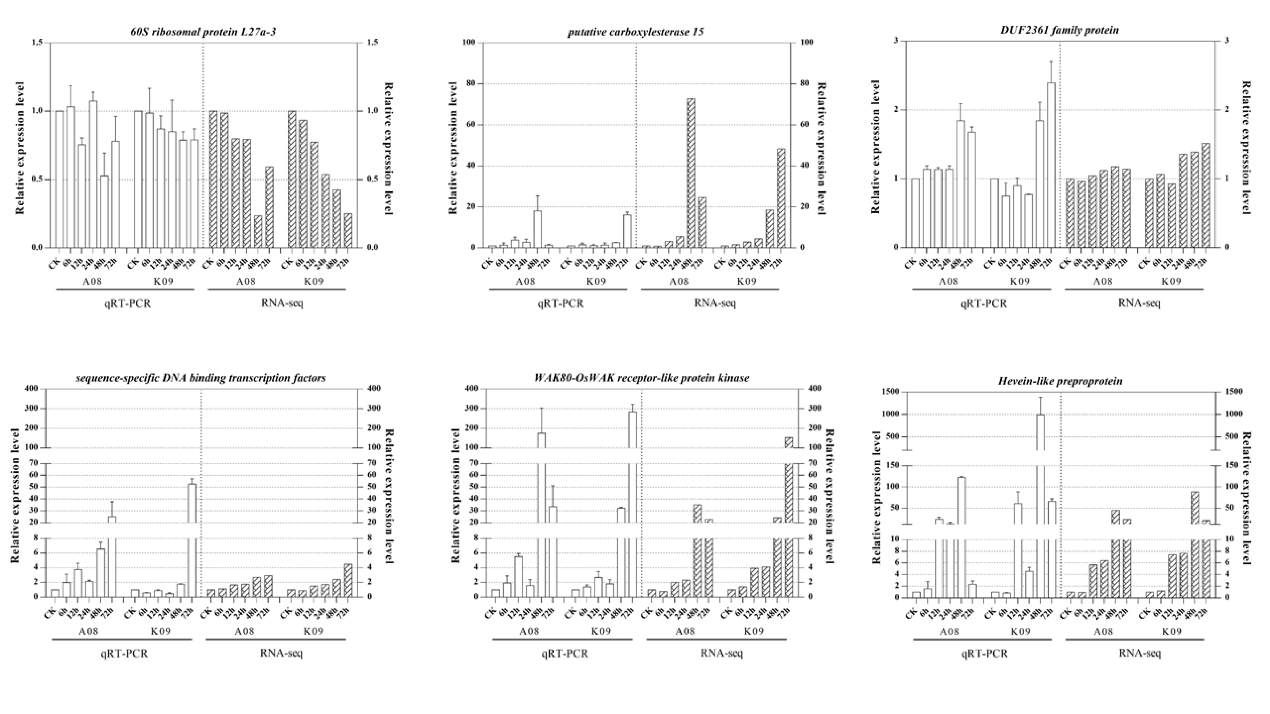
**

**Supplemental Figure S5.** Validation of RNA-seq by qRT-PCR. The genes randomly selected for validation were labeled correspondingly on the top of each panel.


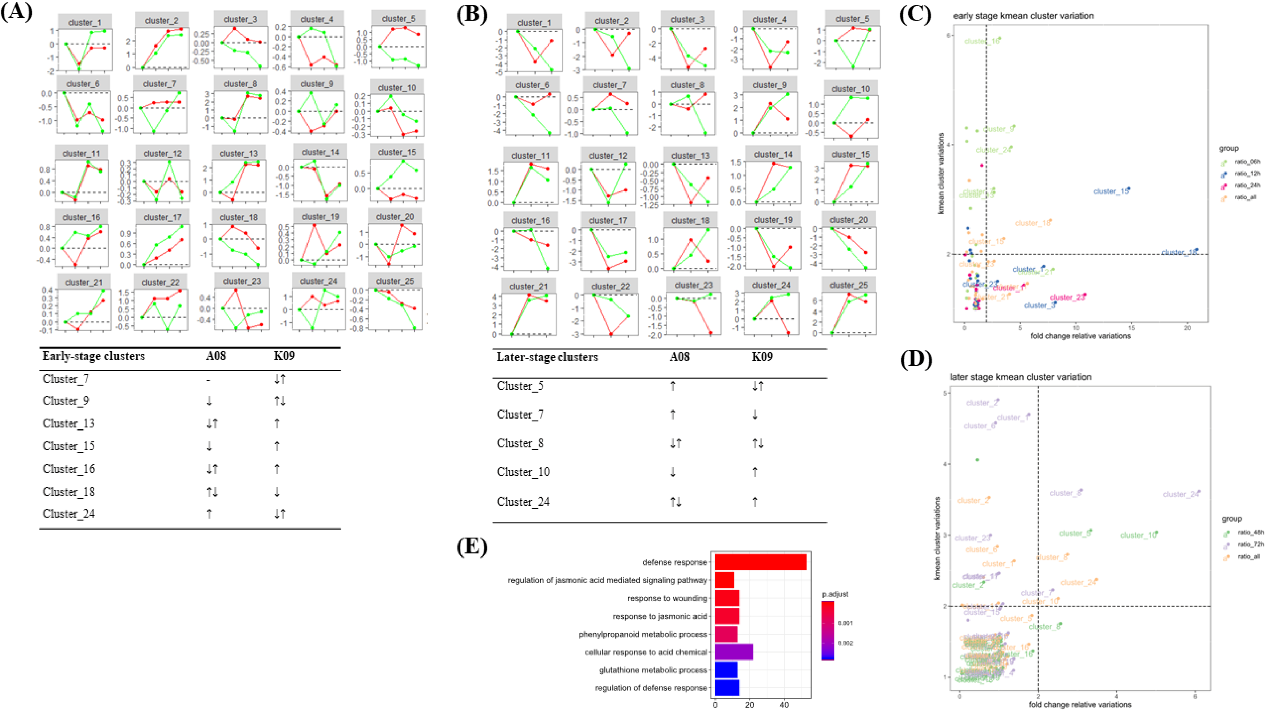


S**upplemental Figure S6.** K-means clustering analysis of DEGs identified in A08 and K09. (**A**) early-stage k-means clustering. The table on the bottom indicates the clusters displaying obviously different expression pattern between two lines. (**B**) later-stage k-means cluster. The table on the bottom indicates the clusters displaying obviously different expression pattern between two lines. (**C**) the ratio of relative standard deviation within susceptible/resistance over relative standard deviation in total at early stages. **(D)** the ratio of relative standard deviation within susceptible/resistance over relative standard deviation in total at later stages. (E) GO biological process enrichment of 407 genes display different expression pattern in resistance and susceptible samples at both early and later stages.


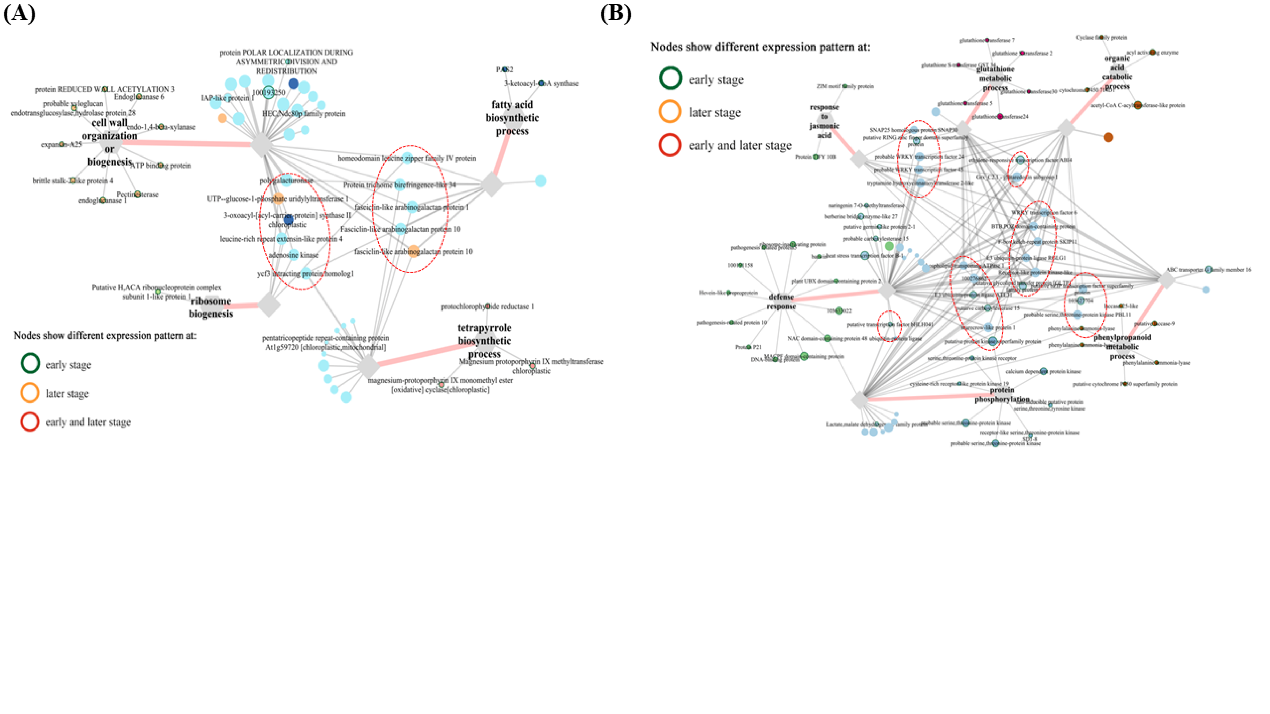


**Supplemental Figure S7**. Network of top 5 “hub genes” co-expressed with pray genes, which showed different expression pattern at early stage between two lines, in (**A**) turquoise module and (**B**) blue module. Hexagons on the graph represent pray genes grouped by GO terms, and each node linked with hexagons represents genes in corresponding GO term, and nodes linked with diamond represent genes co-expressed with pray genes grouped by GO terms. Genes in red dashed ellipse represent “hub genes” that co-expressed with more than one group of pray genes, and nodes with colored border indicate different expression patterns between two lines.


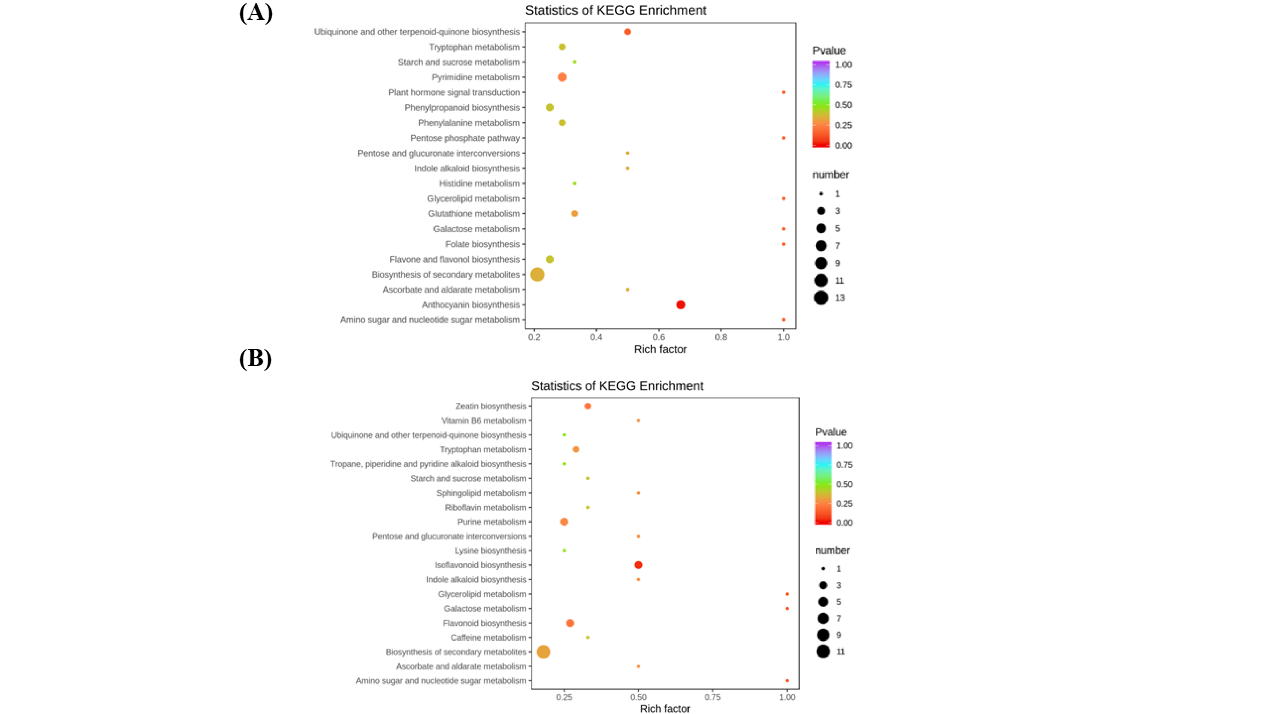


**Supplemental Fig S8.** The KEGG pathway enrichment of DAMs in (A) K09 and (B) A08. The size of dot indicates the number of discriminative metabolites identified, and the color represents the p-value of significance.


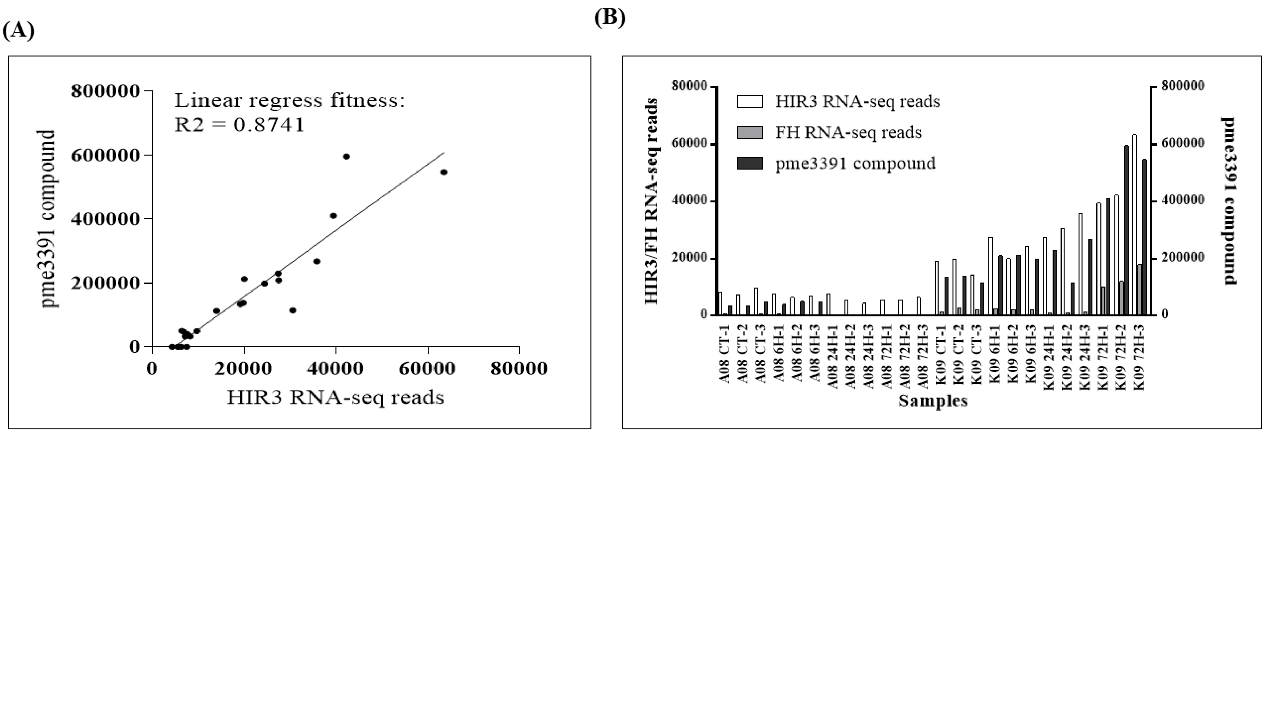


**Supplemental Figure S9.** The correlation between *ZmHIR3* expression level and the compound petunidin 3-O-glucoside (pme3391). (**A**) The linear correlation between *ZmHIR3* RNA-seq counts and the quantification of petunidin 3-O-glucoside (pme3391) by LC-MS/MS. (B) Comparison among *ZmHIR3* RNA-seq reads, fungal RNA-seq reads and the quantification of petunidin 3-O-glucoside (pme3391).


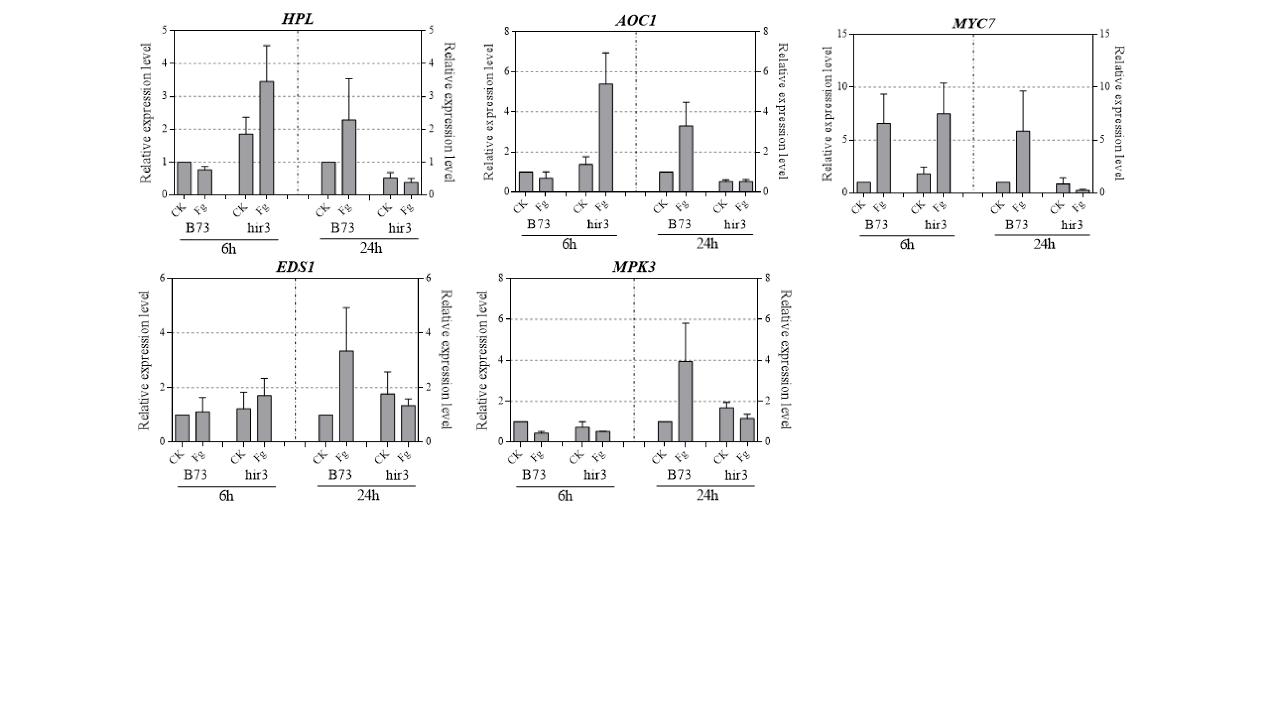


**Supplemental Figure S10.** Quantification of expression levels of selected immune genes in A08 and K09 at 6 hpi and 24 hpi upon *F. graminearum* infection.
