# Supplementary material for: Integrated Gene Co-expression Analysis and Metabolites Profiling Highlight the Important Role of ZmHIR3 in Maize Resistance to Gibberella Stalk Rot
Source: Front Plant Sci. 2021 May 11;12:664733. doi: 10.3389/fpls.2021.664733 (PMC8144520; doi:10.3389/fpls.2021.664733)
Supplement: Supplementary file 2 [file Data_Sheet_2.docx]

**Supplemental Table S1**. Primers used in this study.

| **Gene ID** | **Gene annotation** | **Forward primer (5'to3')** | **Reverse primer (5'to3')** |
| --- | --- | --- | --- |
| **zmhir3 mutant resequencing primers** | | | |
| Zm00001d039173 | zmhir3 | CCGTTGACTTCAGCTATGG | CTGTACGGACGACATAGTCAC |
| **qRT-PCR primers** | | | |
| Zm00001d022124 | 60S ribosomal protein L27a-3 | GACAAGTACCATCCGGGCTA | GCACCTTGAAGTAGCCGAAC |
| Zm00001d013099 | putative carboxylesterase 15 | ATGTCGAGTTCGAGGGTCAG | CCATACACGAAGCGCTTGAC |
| Zm00001d013072 | DUF2361 family protein | AAGGACCTTCCGGATGAGAT | CACCATTTGAACGTTGTTGC |
| Zm00001d048950 | Hevein-like preproprotein | CGGCGACAACTGAACTGATA | AGAACGGACAACGGAGGTAG |
| Zm00001d006331 | sequence-specific DNA binding transcription factors | GTTCGCTGCTTTTGGTCAAT | GCTCAACGGCTTGCTTTATC |
| Zm00001d020955 | WAK80-OsWAK receptor-like protein kinase | AACGCGTGGGTCGACTATAC | AGCATGAGCCGTTCTTCAGT |
| Zm00001d054067 | HPL | TGTACCTCGACACGCAGGAG | CGACGGCAGCGACAAGAAG |
| Zm00001d029594 | AOC1 | ACGTGTACGAGATCAACGAG | TGCTGGATGAGCACGCAGATG |
| Zm00001d030028 | MYC7 | CAGCAGAGGCTACAAGCCAT | CGTCGCAGCCCTTGTAGTAG |
| Zm00001d005708 | EDS1 | CCTTCCTCGGGAACTTCCGT | CCTCCTGATGAATGGCCTGT |
| Zm00001d028711 | MPK3 | CCCAGTTTCCGATATTAGAGATG | GCAGCGACAGATTACACG |
| Zm00001d012277 | *Actin* | CCTTCATTGGCATGGAATCT | GCAACCACCTTCACCTTCAT |
| **ZmHIR3-pCambia1305.1-GFP cloning primers** | | | |
| Zm00001d039173 | *ZmHIR3* | CAGCCCAGATCAACTAGTATGGACTCCTGCTTTCACGTTCTC | CTTGCTCACCATGGATCCGTGTTTGGCGACAGAGGATCCCTG |

**Supplemental Table S2**. Correlation coefficient between field and indoor GSR phenotypes

| Group | Group size (n) | Correlation coefficient | P Value |
| --- | --- | --- | --- |
| All | 41 | 0.0289 | 0.8576 |
| MR+MS | 24 | -0.6022 | 0.0018** |
| HR+HS | 17 | 0.7778 | 0.0002*** |

Note: All, all inbred lines having both field and the indoor phenotypes. MR, the inbred lines displaying medium resistance in both conditions. MS, the inbred lines displaying medium susceptibility in both conditions. HR, the inbred lines displaying high resistance in both conditions. HS, the inbred lines displaying high susceptibility in both conditions. ** and *** represent the significance with p<0.05 and p<0.001, respectively.

**Supplemental Table S3**. Mapping rates of RNA-seq samples. CT, control; 6H, 12H, 24H, 48H, 72H represent hours post inoculation with *Fusarium* *graminearum,* respectively. The number following the treatment stands for the biological replicates.

| Sample | Total reads | Mapping rate | Sample | Total reads | Mapping rate |
| --- | --- | --- | --- | --- | --- |
| A08_CT_1 | 26167786 | 90.87% | K09_CT_1 | 36505610 | 91.69% |
| A08_CT_2 | 34433579 | 90.42% | K09_CT_2 | 28418642 | 91.88% |
| A08_CT_3 | 22262831 | 89.65% | K09_CT_3 | 25729897 | 91.38% |
| A08_CT_4 | 42216978 | 88.90% | K09_CT_4 | 22907118 | 89.96% |
| A08_CT_5 | 25703712 | 91.20% | K09_CT_5 | 21322565 | 90.21% |
| A08_CT_6 | 28679922 | 90.42% | K09_CT_6 | 29351151 | 90.41% |
| A08_6H_1 | 27164907 | 90.73% | K09_CT_7 | 20942695 | 91.05% |
| A08_6H_2 | 26404939 | 90.81% | K09_6H_1 | 31844906 | 91.90% |
| A08_6H_3 | 30618594 | 90.75% | K09_6H_2 | 24480553 | 91.99% |
| A08_12H_1 | 20764382 | 88.52% | K09_6H_3 | 27667938 | 91.29% |
| A08_12H_2 | 27045758 | 90.09% | K09_12H_1 | 23684224 | 89.11% |
| A08_12H_3 | 27028042 | 90.07% | K09_12H_2 | 15078016 | 91.33% |
| A08_24H_1 | 19296896 | 90.82% | K09_12H_3 | 24634120 | 91.33% |
| A08_24H_2 | 23664452 | 89.96% | K09_24H_1 | 28645189 | 91.16% |
| A08_24H_3 | 25734193 | 90.05% | K09_24H_2 | 23783846 | 89.66% |
| A08_48H_1 | 24072076 | 83.08% | K09_24H_3 | 27175079 | 91.92% |
| A08_48H_2 | 19893345 | 83.94% | K09_48H_1 | 20001329 | 88.69% |
| A08_48H_3 | 27310937 | 85.84% | K09_48H_2 | 24989369 | 89.16% |
| A08_72H_1 | 26954311 | 86.46% | K09_48H_3 | 33077543 | 88.01% |
| A08_72H_2 | 26670870 | 87.19% | K09_72H_1 | 20118419 | 79.16% |
| A08_72H_3 | 28214618 | 86.56% | K09_72H_2 | 21682109 | 79.00% |
|  |  |  | K09_72H_3 | 30678950 | 84.18% |

**Supplemental Table S4.** Pearson correlation of pme3391(Petunidin 3-O-glucoside) with the expression of highly correlated genes

|  | **Gene_id** | **Description** | **Pearson correlation** |
| --- | --- | --- | --- |
| Top 10 positive correlated genes | Zm00001d033110 | inositol monophosphatase 3 | 0.954 |
|  | Zm00001d028286 | serine,threonine receptor-like kinase NFP | 0.948 |
|  | Zm00001d031954 | Zm00001d031954 | 0.943 |
|  | Zm00001d037556 | Alpha-L-fucosidase 2 | 0.942 |
|  | Zm00001d038050 | Zm00001d038050 | 0.937 |
|  | Zm00001d043258 | caltractin | 0.936 |
|  | Zm00001d039173 | hypersensitive induced reaction 3 | 0.936 |
|  | Zm00001d012975 | glycine-rich protein A3 | 0.935 |
|  | Zm00001d029194 | isoflavone 2'-hydroxylase | 0.935 |
|  | Zm00001d003357 | Copper transport protein family | 0.935 |
| Top 10 negative correlated genes | Zm00001d050838 | polyadenylate-binding protein RBP47 | -0.959 |
|  | Zm00001d049228 | Putative membrane lipoprotein | -0.95 |
|  | Zm00001d007031 | 10 kDa chaperonin[mitochondrial] | -0.948 |
|  | Zm00001d012158 | formin-like protein 1 | -0.946 |
|  | Zm00001d026547 | trafficking protein particle complex subunit 3 | -0.945 |
|  | Zm00001d012578 | DNA-3-methyladenine glycosylase I | -0.945 |
|  | Zm00001d019139 | sterol methyltransferase1 | -0.943 |
|  | Zm00001d023343 | non-specific lipid-transfer protein | -0.942 |
|  | Zm00001d020695 | nucleoporin p58,p45 | -0.941 |
|  | Zm00001d016164 | GW2 | -0.935 |
